# Laser-Induced Graphitization of Polydopamine on Titania Nanotubes

**DOI:** 10.1021/acsami.3c11580

**Published:** 2023-11-02

**Authors:** Adrian Olejnik, Krzysztof Polaczek, Marek Szkodo, Alicja Stanisławska, Jacek Ryl, Katarzyna Siuzdak

**Affiliations:** † Department of Metrology and Optoelectronics, Faculty of Electronics, Telecommunications and Informatics, Gdańsk University of Technology, Narutowicza 11/12 St., Gdańsk 80-233, Poland; ‡ Centre for Plasma and Laser Engineering, The Szewalski Institute of Fluid-Flow Machinery, Polish Academy of Sciences, Fiszera 14 St., Gdańsk 80-231, Poland; § Department of Biomedical Chemistry, Faculty of Chemistry University of Gdansk, Wita Stwosza 63 St, Gdańsk 80-308, Poland; ∥ Institute of Manufacturing and Materials Technology, Faculty of Mechanical Engineering and Ship Technology, Gdańsk University of Technology, Narutowicza 11/12 St., Gdańsk 80-233, Poland; ⊥ Institute of Nanotechnology and Materials Engineering and Advanced Materials Center, Gdańsk University of Technology, Narutowicza 11/12, Gdańsk 80-233, Poland

**Keywords:** polydopamine, graphitization, laser-induced graphene, titania nanotubes, photoelectrochemistry, intensity-modulated photovoltage spectroscopy, IPCE

## Abstract

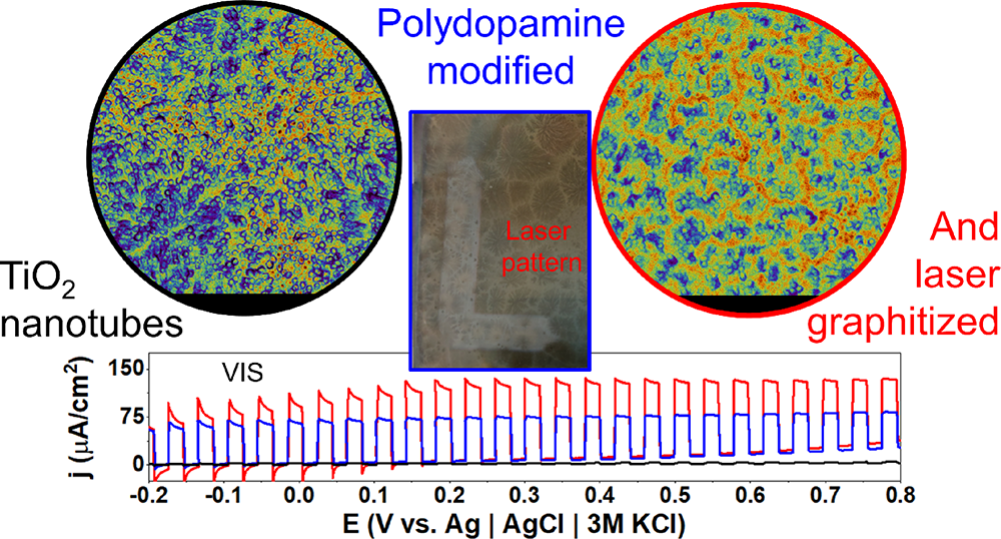

Since the discovery of laser-induced graphite/graphene, there has been a notable surge of scientific interest in advancing diverse methodologies for their synthesis and applications. This study focuses on the utilization of a pulsed Nd:YAG laser to achieve graphitization of polydopamine (PDA) deposited on the surface of titania nanotubes. The partial graphitization is corroborated through Raman and XPS spectroscopies and supported by water contact angle, nanomechanical, and electrochemical measurements. Reactive molecular dynamics simulations confirm the possibility of graphitization in the nanosecond time scale with the evolution of NH_3_, H_2_O, and CO_2_ gases. A thorough exploration of the lasing parameter space (wavelength, pulse energy, and number of pulses) was conducted with the aim of improving either electrochemical activity or photocurrent generation. Whereas the 532 nm laser pulses interacted mostly with the PDA coating, the 365 nm pulses were absorbed by both PDA and the substrate nanotubes, leading to a higher graphitization degree. The majority of the photocurrent and quantum efficiency enhancement is observed in the visible light between 400 and 550 nm. The proposed composite is applied as a photoelectrochemical (PEC) sensor of serotonin in nanomolar concentrations. Because of the suppressed recombination and facilitated charge transfer caused by the laser graphitization, the proposed composite exhibits significantly enhanced PEC performance. In the sensing application, it showed superior sensitivity and a limit of detection competitive with nonprecious metal materials

## 1 Introduction

Polydopamine (PDA) and melanin-based materials represent a vast research topic in electrochemistry photochemistry, surface science, and electronics with many promising applications in each field.^1−3^ Despite its intrinsically rich chemistry^4−6^—associated with the amine–catechol–quinone interplay—and possibilities for various physical structures including π–π stacking, π-cation, or aryl–aryl linking^6−8^ there are still numerous procedures for altering its structure on various levels. Those procedures can include monomer alteration at the synthesis level (adrenaline, noradrenaline, l-DOPA, tyrosine derivatives, and more)^9−11^ or direct modification of functional groups including amination, carboxylation,^12^ and recently also N-methylation.^13^ Alternatively, it is possible to include other chemical entities inside the PDA matrix such as transition metals^14,15^ or organic molecules during the course of either oxidative or electropolymerization.^16−18^ Proposed modifications are aimed at an incredibly vast scope of applications such as supercapacitors,^19^ protection from photocorrosion,^20^ generation of fluorescence,^21^ flexible electronics,^2^ and biomedicine including biosensing; PDA can act as an element of the organic layer onto the electrode^18,22^ or as an imprinted polymer.^23^

One of the most serious problems limiting applications is poor mechanical properties associated with high roughness and agglomeration. This problem is especially urgent in the field of electrochemical applications^19^ and colorimetric sensing.^24^ An idea for improvement in this area was proposed in a *Nature Communications* paper in 2020^25^ via the laser-graphitization protocol of the PDA. In this work, PDA was deposited on quartz using standard oxidative polymerization in Tris buffer. Then, it was detached from the surface and treated with a blue diode continuous laser with 1–2 W power. Through XPS and Raman measurements, it was evidenced that partial graphitization occurs with covalent coupling between the PDA units. The modification resulted in a 100-fold increase in scratch resistance, which was a higher value than quartz and TiO_2_ reference parameters. The initial high-roughness layer was changed to a uniform 37 nm film without agglomerated PDA nanoparticles. More importantly, however, the most desired properties of PDA such as high adhesiveness and biofouling resistance associated with the catechol functionality were preserved.

The laser-graphitized PDA (lgPDA) can be considered a member of the laser-induced graphene/graphite (LIG) class of materials, which is prominent for electronic and electrochemical applications.^26−28^ In general, the purpose of the LIG-ation is to change the weakly conducting π-conjugated organic polymer such as polyether(ether–ketone) or poly(etherimide) into a highly conductive carbon-based sheet, typically N-doped graphene/graphite. From an electrochemical point of view, such a treatment facilitates charge transfer at the electrode|electrolyte interface. In general, LIG finds many applications including electrochemical sensing, supercapacitors, electrocatalysis, and photodetectors.^29,30^

Besides laser treatment, there are several other approaches for synthesis of graphite/graphene and other nanocarbons from organic precursors, such as polymers, resins, or biomass. These include various high-temperature and chemical treatments, but for simplicity, they can be divided into catalytic graphitization/carbonization/pyrolysis and hydrothermal treatment.^31^

There are two distinguishable trends in the related literature. The first one is aimed at biomass conversion toward cheap and large-scale production of nanocarbons. In this space, reduction of the graphitization temperature, high output, simplicity, and stability of the fabrication method are highly appreciated.

Development of graphitization catalysts and dispersion agents for precursors^32^ allows progress in the field. There are many types of catalysts including transition metals (Fe, Ni, Co),^33^ their salts and oxides,^34^ boron compounds,^35^ and carbon nanostructures.^36,37^ Among them, iron-catalyzed graphitization is an exemplary solution for carbonization of various kinds of precursors, and many protocols are already developed.^38^ Despite the efforts, the pyrolysis methods require temperatures above 1000 °C and even up to 3000 °C and an inert atmosphere for efficient conversion.^31,33^

In contrast to the pyrolysis approaches, hydrothermal process is based on autoclaving the pretreated precursors at lower temperatures in alkaline or acidic environments.^31^ However, the carbonized product is typically strongly disordered and oxidized and requires another high-temperature step to transform into crystalline nanocarbons, such as graphene. For example, Chen et al. applied such methodology for wheat straw waste.^39^ The temperature required for graphitization was as high as 2600 °C to achieve a quality sufficient for electrochemical applications.

The second trend in the graphitization literature is focused on the modification of functional nanomaterials. In this space, fine-tuning of the electrical, thermal, and mechanical properties toward various applications is highly desired. Almost all of the work concerning PDA and its electrochemical applications falls into this category. The typical method of PDA graphitization/carbonization is purely thermal and requires temperature values between 700 and 1000 °C.^40−43^ The resulting carbonized PDA possesses the structure of nitrogen-doped graphite with varying levels of crystallinity and ordering. Surprisingly, one work reports the calcination of PDA to amorphous carbon at relatively low 500 °C in a nitrogen flow.^44^ On the other hand, in some carbon–carbon composites, temperatures as high as 3000 °C are required.^45,46^

Thus, the current graphitization state of the art renders LIG-ation as an appealing, energy-efficient alternative because of the markedly lower energy dissipation. An ambient atmosphere is typically also sufficient.^47^ Surprisingly though, according to the best knowledge of the authors, there have been no further realizations of the PDA laser modifications or LIG-ation in the literature since the *Nature Communications* paper in 2020. This idea is strongly potent not only in the context of PDA mechanical properties and expanding its library of chemical structures but also for elucidation of its complicated electronic structure.

PDA can be synthesized in the form of a free-standing film^48^ or nanoparticles^46,49^ as an element in composition with different materials (metals, semiconductors, organics).^50^ In the seminal paper^25^ describing laser graphitization of the PDA for the first time, PDA was in the form of a film. However, laser treatment of the PDA bound to some surface has not been studied yet, and the influence of such treatment on various properties of this composite material is unknown. Moreover, the electronic structure of the PDA–substrate composite will be clearly altered by laser treatment with various parameters.

In particular, PDA–semiconductor interfaces represent an especially interesting subgroup of the PDA–substrate composites due to nontrivial electrical and electrochemical properties.^51−54^ In general, PDA can be viewed as an amorphous semiconductor (due to temperature dependence of the conductivity) with the ionic component of the conduction.^50^ On the other hand, in contact with other semiconductors, it acts as a photosensitizer or another semiconductor depending on the film thickness.^51,54,55^

In our previous work, we have shown the alternative picture to think of quinone-rich PDA as a semimetallic entity. Such PDA electropolymerized on the surface of titania nanotubes leads to 20-fold photocurrent enhancement through photosensitization as it behaves as a net of spatially distributed redox mediators in electrochemical experiments with redox mediators.^56^

In this work, electropolymerized PDA is modified using a pulsed Nd:YAG laser with 365 and 532 nm wavelengths and varying pulse parameters. Titania nanotubes are chosen as a substrate for PDA deposition (prelaser graphitization) for several reasons. First, TiO_2_ is a well-studied semiconductor with a high band gap and is prone to photosensitization with dyes or PDA.^54,56,57^ Second, nanotubes exhibit nanomicroporous morphology resulting in a high PDA loading. Therefore, it is a reasonable platform to study the PDA–semiconductor interactions. Partial graphitization is confirmed by several techniques, including XPS, Raman scattering, nanoindentation, water contact angle, and electrochemical measurements. Graphitization is modeled on the molecular level by a reactive molecular dynamics approach. Wavelength-resolved photoelectrochemistry and quantum efficiency measurements are employed to elucidate the mechanism of enhanced absorption and photocurrent generation in the visible range.

## 2 Materials and Methods

### 2.1 Hydrogenation of TiO_2_ Nanotubes and Electropolymerization of Dopamine

The list of chemicals and protocol for titania nanotube (TNT) synthesis can be found in the SI file. All electrochemical experiments, including the hydrogenation and electropolymerization of dopamine, were performed by using a Biologic SP-150 potentiostat–galvanostat.

Unless stated otherwise, the working electrode was hydrogenated TNT (pure or modified), the counter electrode was a platinum mesh, and the reference electrode was Ag|AgCl|3 M KCl. All potentials in this article are expressed with respect to this reference electrode. The geometric surface areas of the working electrodes were between 0.5 and 1 cm^2.^

Hydrogenation of titania nanotubes was performed in an argon-purged 0.5 M Na_2_SO_4_ solution through the following protocol. First, linear sweep voltammetry (LSV) with a 100 mV/s rate was used for cathodic polarization up to −5 V. Then, a constant potential equal to −5 V was applied for 2 min.

Electropolymerization of dopamine on hydrogenated TNT electrodes was carried out potentiodynamically in an argon-purged solution of 0.5 M Na_2_SO_4_ containing 1× Tris buffer immediately after hydrogenation. The pH of the solution was adjusted by adding 1 M HCl or 1 M NaOH to the desired values, i.e., 5.7, 7.5, 8.5, 9.0, or 10.0. An MP-103 handheld potentiometric pH meter was used to control the pH value. The number of CV cycles was 5, 10, 25, or 50 with a 20 mV/s scan rate in the range from −0.5 to +1.0 V.

### 2.2 Laser Modification of the PDA-Modified Nanotubes

Laser modification of samples aimed at PDA graphitization was performed using Nd:YAG (QSmart 850 Quantel) pulsed nanosecond laser using second (532 nm) and third (365 nm) harmonics. The pulse frequency was set to 10 Hz, and energy fluences varied between 30 and 120 mJ/cm^2^ abbreviated as F30, F60, F90, and F120. Beam homogenizers for both wavelengths were used to maintain a flat profile of the light intensity during exposure. Electrodes were lased in the programmed rectangular shape of a ca. 1 cm^2^ area using the motorized table. The variable velocity of the table allows a variable number of laser pulses the sample is exposed to. In particular, speeds S200, S100, S50, and S25 correspond to 200, 100, 50, and 25 cm/h, respectively. Given that the spot for the 365 nm modification is a square with a 2.6 mm side, these would correspond to 4.6, 9.2, 18.4, and 36.8 pulses, respectively, for each point on the sample. Analogously, for the spot used for 532 nm modification, these would correspond to 6.4, 12.8, 25.6, and 51.2 pulses per sample point on average. All treatments were performed in a vacuum of 10^–4^ mbar to mitigate the possible influence of oxygen and carbon dioxide present in the air.

### 2.3 SEM Inspection and Measurement of the PDA Film Thickness

The surface morphology of the electrodes was investigated using a Quanta FEG 250 (FEI) Schottky field-emission scanning electron microscope (SEM) equipped with a secondary ET electron detector with a beam accelerating voltage of 10 kV. The thickness of the PDA layer (*d*_PDA_) was estimated by measuring the wall thickness of the PDA-modified nanotubes (*W*_TNT|PDA_) and then subtracting the thickness of the unmodified nanotubes (*W*_TNT_) and dividing by 2 (eq 1):
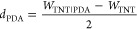
1

### 2.4 Nanoindentation

The nanoindentation test was performed on the surface of TNT nanotubes as well as nanotubes with dopamine deposited on them (TNT_PDA) and dopamine modified with laser (TNT_IgPDA). The tests were conducted by using a Berkovich indenter with a maximum load of 0.4 mN. The loading rate of the indenter was 0.1 mN/s. Once the maximum load was reached, it was maintained for 5 s, and then the load was removed at a rate of 0.1 mN/s. The subsequent measurements were taken at the same location, with the described cycle repeated 10 times. After each cycle, the hardness and stiffness of the surface were determined using the Oliver–Pharr method.^58^

The elastic properties of the surface were also characterized by the elastic work during the unloading of the indenter. This work was calculated as the area under the unloading curve of the indenter, i.e., under the curve *P = f(h)*, where *P* is the indenter load and *h* is its displacement in the tested material. Both hardness and stiffness, as well as the elastic work, were calculated by using the NanoTest Ventage nanoindenter software.

In each load cycle, the creep rate of the nanotubes at a constant maximum load was also determined. The creep rate was calculated based on the Δ*h/h =* ε *= f(t)* curve, where Δ*h* is the depth of penetration increment of the indenter at a constant maximum load and *t* is the time at a constant maximum load. The creep rate dε/d*t* was determined for a steady-state condition in which Δ*h/h* was a linear function of time *t*.

### 2.5 Electrochemical and Photoelectrochemical Characterization

Photoelectrochemical tests were performed in 0.5 M Na_2_SO_4_ electrolyte using linear square voltammetry (LSV) with a scan rate of 20 mV/s or using chronoamperometry (CA) at a potential of +0.3 V. As a light source, a xenon lamp equipped with AM 1.5 and UV cutoff (GG40, Schott) filters were used. The irradiation intensity was established to be 100 mW/cm^2^ using a Si reference cell (Rera).

Photocurrent action maps were measured with a photoelectric spectrometer for quantum efficiency measurements (Instytut Fotonowy, Poland) equipped with a solar simulator, Czerny–Turner monochromator, and potentiostat. The illumination source was calibrated by using a silicon photodiode to calculate light intensities. The wavelength range was set from 200 to 700 nm, and the potential range was set from 100 to 800 mV vs Ag|AgCl|3 M KCl reference electrode. Measurement points were taken with 25 nm and 50 mV steps.

External quantum efficiencies of photocurrent generation (or IPCE = incident photon to current efficiencies) were calculated for several wavelengths (373, 398, 424, 455, and 524 nm) and with variable light intensities using LED illumination sources. The standard formula was applied:
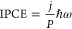
2where *j* is the photocurrent, *P* is light intensity, and ℏω is photon energy.

Intensity modulated photocurrent/photovoltage spectroscopy (IMPS/IMVS) experiments were performed on a dedicated device provided by the Instytut Fotonowy equipped with a revolver containing several LEDs with adjustable AC and DC components of the illumination and a potentiostat.

IMPS measurements were carried out with 373 and 424 nm LED wavelengths with variable AD and DC components indicated in the text. The sample was polarized with a + 500 mV anodic bias (with respect to the Ag|AgCl|3 M KCl reference electrode). The applied frequency range was 100 mHz–10 kHz, and for each frequency, two measurement points were registered. IMVS measurements were carried out with 373 nm LED with 0.2 mW/cm^2^ AC and 1 mW/cm^2^ DC components of the illumination in open circuit conditions.

### 2.6 ReaxFF Simulation of PDA Graphitization

Molecular structures of PDA units were designed using a builder tool provided by Atomistix ToolKit Quantumwise (ATK, Synopsys, USA) as reported in ref (59). The process of PDA graphitization was simulated using a reactive force field (ReaxFF) molecular dynamics (MD) approach.^60,61^ The initial structure for the MD trajectory was a DFT-optimized set of four PDA tetramers based on DHI units. The density of molecules was set to 0.6 g/cm^3^ with periodic boundary conditions similarly as proposed in a previous work^62^ devoted to laser graphitization of other analogous polymers. An NVT Nosé-Hoover thermostat with 0.25 fs time step and three elements in Nosé-Hoover chains were used for generating MD trajectories up to 4 ns. Gases evolving throughout the simulation were kept in the box. Temperatures of 2000, 3000, and 4000 K were tested, but results are only presented for 3000 K because no significant temperature dependence on the final structure was observed. Analysis of MD trajectories was performed with an analyzer tool available in the ATK package as implemented.^63^ The resulting lgPDA structure was then used for DFT calculations of the band structure and density of states; details can be found in the SI file.

### 2.7 Photoelectrochemical (PEC) Detection of Serotonin

Photoelectrochemical (PEC) sensing experiments were conducted using the cell and light sources identical to those in the case of the IMPS and quantum efficiency measurements. The light source was a 373 nm LED with 2 mW/cm^2^ light intensity switched on/off during the chronoamperometirc polarization equal to +100 mV.

Prior to the main sensing experiment, the sample was preconditioned in several ways. First, five CV cycles were applied in the range between −0.2 and +0.8 V and 100 mV/s scan rate. The purpose was to achieve surface reconstruction and remove all the residual currents that could potentially interfere with the proper analyte signal. Second, the gradual light exposure, i.e., 0.5, 1, 1.5, and 2 mW/cm^2^, was applied at −100 mV (it is ca. an open circuit potential). Third, the sample was polarized to +100 mV and held for 20 s until the second, analogous, gradual light exposure was applied. The current response of the final step at +100 mV and 2 mW/cm^2^ is shown and used for construction of calibration curves. Limits of detection were calculated according to ref (64) as three standard deviations divided by the slope of the linear range.

The sample chosen for this experiment was TNT_lgPDA_365 synthesized using 365 nm laser, 60 mJ/cm^2^ fluence, and S25 table speed. The surface area exposed to light was equal to 0.785 cm^2^.

## 3 Results and Discussion

### 3.1 General Synthesis Protocol for the Laser Graphitized Polydopamine (lgPDA) on the Surface of Titania Nanotubes (TNTs)

**Figure 1:**
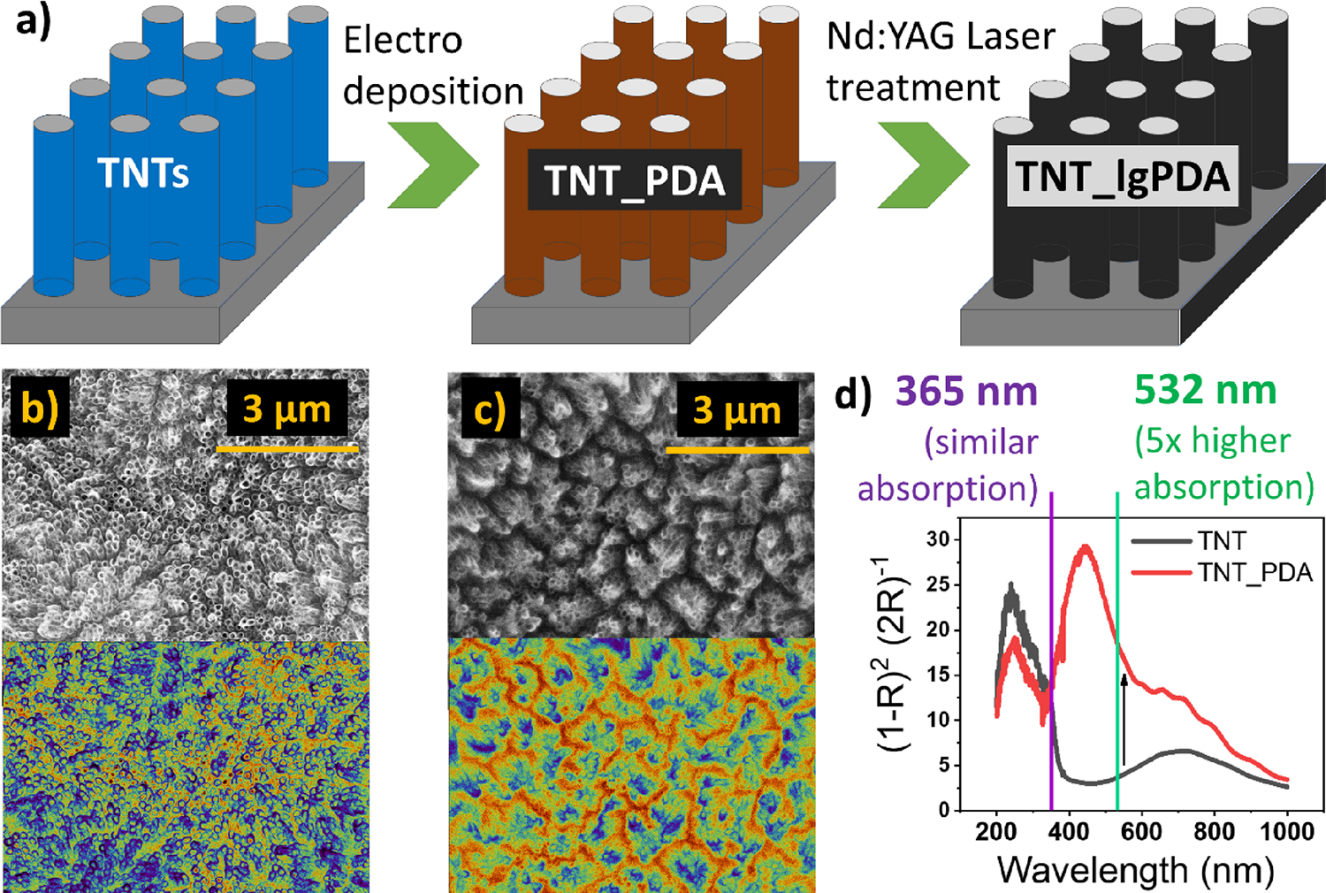
(a) Synthesis of the laser graphitized polydopamine (lgPDA) on titania nanotubes (TNT). (b) SEM inspection of the pristine TNT. (c) After electropolymerization of PDA, two false-color contrasts of the same images are shown. (d) Kubelka–Munk functions obtained from diffuse reflectance data of pristine and PDA-modified TNTs with denoted wavelengths of the Nd:YAG laser used for treatment.

In general, the synthesis protocol adopted in the following study involved the anodization of titanium foil to obtain crystalline titania nanotubes (TNTs) with various geometries. Then, TNTs were electrochemically hydrogenated, and polydopamine (PDA) was deposited on them by electropolymerization of dopamine according to the procedure described in our previous works.^16,65^ An exemplary CV curve of electropolymerization can be found in Figure S1 with characteristic redox peaks originating from reactions of the PDA structural units. The resulting TNT_PDA heterostructure was modified by the nanosecond pulsed Nd:YAG laser using two wavelengths, i.e., 532 and 365 nm (second and third harmonics), to obtain the final laser-graphitized polydopamine (lgPDA). The protocol is schematically presented in Figure 1a with SEM images acquired for the pristine (Figure 1b) and PDA-modified nanotubes (Figure 1c). Chosen wavelengths are based on the optical spectroscopy data of the TNTs and TNT_PDA so that the 532 nm pulse is targeting mostly the absorption by the PDA, whereas the 365 nm pulse should interact both with the TNTs and the PDA (see the Kubelka–Munk function in Figure 1d).

### 3.2 Optimization of TNT and PDA Synthesis Parameters prior to Laser Graphitization

Several geometries of titania nanotubes were synthesized to screen for the optimal template for laser treatment. In terms of spacing between TNTs, adjacent and loosely spaced nanotubes were prepared in ethylene glycol and diethylene glycol, respectively, and in terms of their length, varying anodization times were applied between 15 min and 2 h. SEM images of the adjacent nanotubes are shown in Figure 1b,c, and those of loosely spaced nanotubes are shown in Figure S2a-d. After the deposition of polydopamine, nanotube walls thicken in both cases, and their coronas are slightly aggregated in the adjacent case. Those phenomena are visually easier to grasp by using the spectral contrast applied to SEM images.

There is also a tendency of TNT_PDA based on longer and adjacent nanotubes to exhibit larger values of the current integral during CV sweeps of electropolymerization, indicating higher surface area and the amount of the PDA deposited (Figure S3a). Moreover, higher vis-photocurrent enhancement due to the PDA presence is observed for the longest nanotubes prepared during 2 h anodization (Figure S 3d). TiO_2_ samples obtained during anodization time exceeding 2 h have the property of the morphology switching from nanotubes to a spongy layer after PDA deposition (Figure S4).

It is known that there exists a large variability of the PDA structure and properties depending on the electropolymerization parameters.^9,22,65,66^ Therefore, the influence of the number of polymerization cycles and the applied pH on the vis-photocurrents and electrochemical performance has been briefly tested for the studied set of TNT_PDA samples (Figure S3). Is it easy to see that longer cycling up to 50 potentiodynamic cycles yields higher photocurrents and lower capacitive background accompanied by higher electroactivity expressed as a smaller distance between ferrocyanide redox peaks. Furthermore, PDA obtained at pH = 7.5 exhibits the same two properties in contrast to its more alkaline variants. The latter does not even exhibit clear ferrocyanide redox peaks and has a higher capacitive background current. This behavior stands in line with the literature reports suggesting lower electroactivity of other similar polymers such as *o*-phenylenediamine^67−69^ synthesized in alkaline pH. Although this property might be beneficial in terms of, e.g., constructing molecular imprinted polymers (MIP) for sensing, for photoelectrochemical studies, the charge transfer rate and photocurrent generation are most important.

Therefore, for laser graphitization, adjacent and long (2 h anodization) nanotubes as well as PDA obtained during 50 cycles with solution pH = 7.5 are chosen for laser graphitization and further photoelectrochemical studies. Unless stated otherwise, TNT and PDA abbreviations correspond to those particular samples throughout the paper.

### 3.3 Physical Properties of the TNT_lgPDA Heterostructures

**Figure 2:**
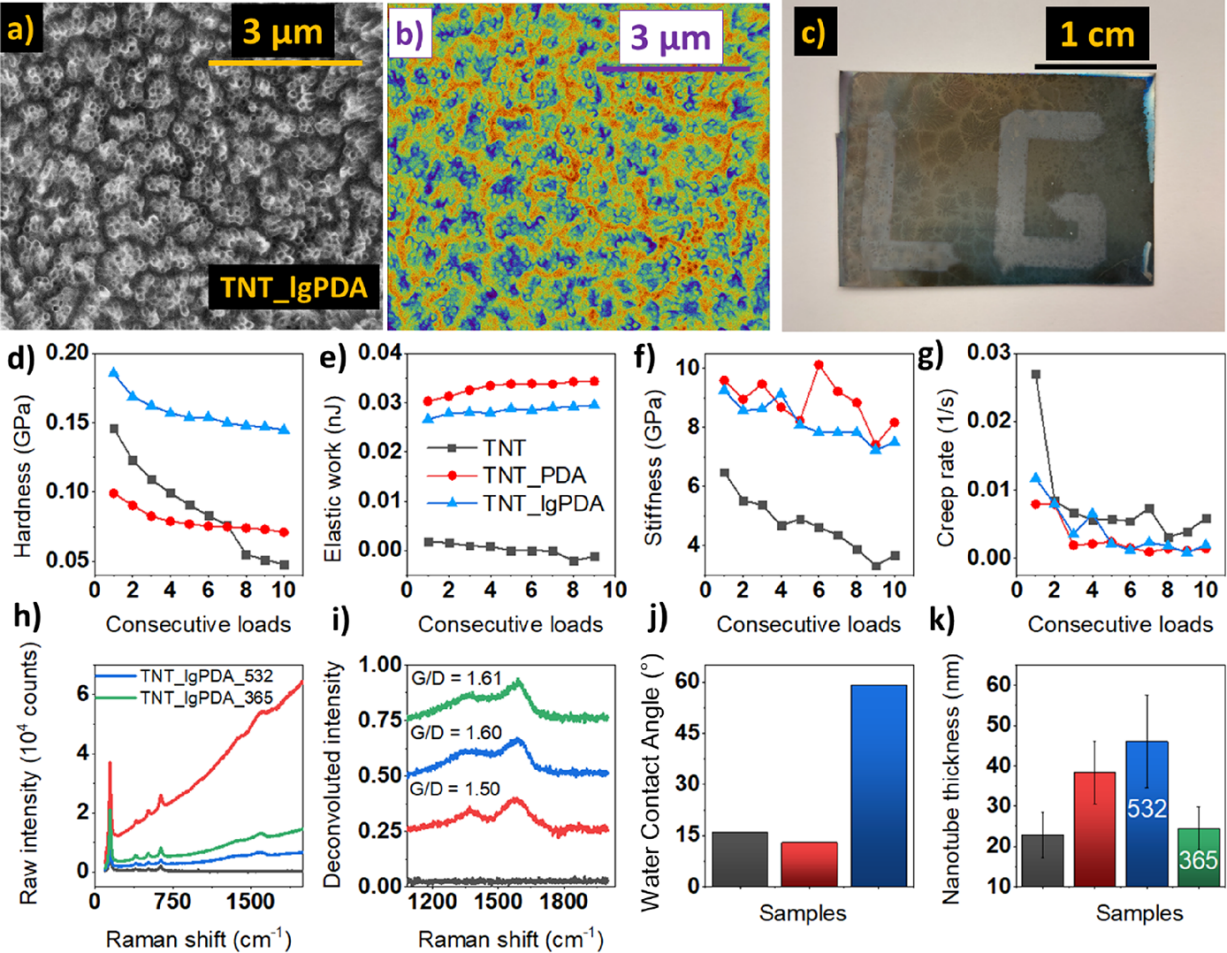
Physical properties of the TNT_lgPDA heterostructure: (a, b) SEM image in two contrasts; (c) macroscopic picture of the laser graphitized sample; (d–g) series of multiload nanoindentation results for hardness, elastic work of deformation, stiffness, and creep rate, respectively; (h) comparison of raw Raman signals for pristine (TNT), PDA-modified (TNT_PDA), and laser graphitized samples (TNT_lgPDA_532, TNT_lgPDA_365); (i) corresponding rescaled Raman signals after subtraction of the fluorescence; (j) water contact angles; and (k) thicknesses of nanotube walls withdrawn from SEM images (images and corresponding statistics can be found in Figures S2 and S7, respectively).

SEM images of the TNT_lgPDA structure are given in Figure 2a and are almost identical to the as-formed TNT_PDA electrode, indicating that laser graphitization does not alter the morphology significantly. This behavior is preserved also for loosely spaced TNTs (Figure S2) using both 532 and 365 nm wavelengths. On the other hand, the macroscopic image of the sample allows easy distinction between the modified and unmodified areas. With the application of the motorized table, spatially selective laser graphitization “lg” is enabled (Figure 2b). It was noted, however, that the treatment via 365 nm with higher fluences (above 90 mJ/cm^2^) leads to a significant distortion of the nanotubes themselves (Figure S5).

Considering that graphitization significantly changes the mechanical properties of the PDA,^25^ a series of repetitive nanoindentation experiments have been performed to validate if the surface-deposited PDA exhibits the same behavior (Figures 2c–f). It is easy to see that hardness of the TNT_lgPDA heterostructure is highest among studied samples, and its decay with consecutive loading is slower with respect to pristine nanotubes. Elastic work during indentation is markedly elevated for both pristine and laser-treated PDA, indicating that the higher energy is required for the deformation to occur. A similar trend is observed also for stiffness (Young’s modulus). Last, the creep rate (i.e., the slope of the deformation with respect to time during the indentation) is decreased after modification with either PDA or lgPDA. Detailed data on each multiload experiment including load–displacement curves and deformation–time curves can be found in Figure S6. In summary, the phenomenon of increased hardness after laser modification has been replicated in the case of surface-deposited PDA. However, its influence on other mechanical properties seems to be negligible in the studied system. Presumably, the PDA itself causes major changes to the TNT electrode mechanical characteristics compared to the subsequent laser treatment.

A clear indication of the presence of PDA on the surface of TNTs is the Raman scattering measurement. In comparison to pristine TNTs, after PDA deposition, a significant increase in the fluorescence is observed accompanied by the emergence of D and G bands at regions around 1350 and 1650 cm^–1^, respectively (Figure 2h). There are also notable changes after laser treatment regardless of the wavelength used, namely, a decrease in the fluorescence signal and an elevation of the G band. The latter can be seen more clearly when the fluorescence baseline is subtracted, and raw Raman data are rescaled (Figure 2i). It can be assumed that the G/D band ratio is a measure of the graphitization degree and will be used in this context throughout the rest of the paper. This ratio is increased from 1.50 to 1.60 after laser treatment, strongly suggesting that graphitization indeed occurred.

Moreover, the water contact angle is also markedly increased from about 15° of pristine TNTs and TNT_PDA to about 60° (Figure 2j). Although the surface remains hydrophilic, the increased water contact angle is another indirect symptom of graphitization. Considering increased hydrophobicity after laser treatment, this solution could be used to facilitate PDA loading of hydrophobic molecules into some porous materials through interactions with lgPDA. That is because even after graphitization, some catechol functionalities still remain.^40^ This could be of strong interest in the fields of wastewater treatment or in colorimetric sensing.^24^

Finally, the thickness of the deposited coating can be roughly estimated from the SEM pictures by comparing the nanotube wall thickness before and after modifications (Figure 2k). It is easy to see that after PDA electropolymerization, there is a 2-fold increase of the thickness up to ca. 40 nm, indicating a 7.7 nm thickness of the PDA. The coating thickens even more to 11.5 nm after laser treatment using a 532 nm wavelength, suggesting the occurrence of the swelling phenomenon. On the other hand, the thickness plummets to 1.1 nm after 365 nm exposure, suggesting that a large part of the PDA is either evaporated or incorporated into the TNT structure after the process.

### 3.4 XPS Measurements of the TNT_lgPDA

**Figure 3:**
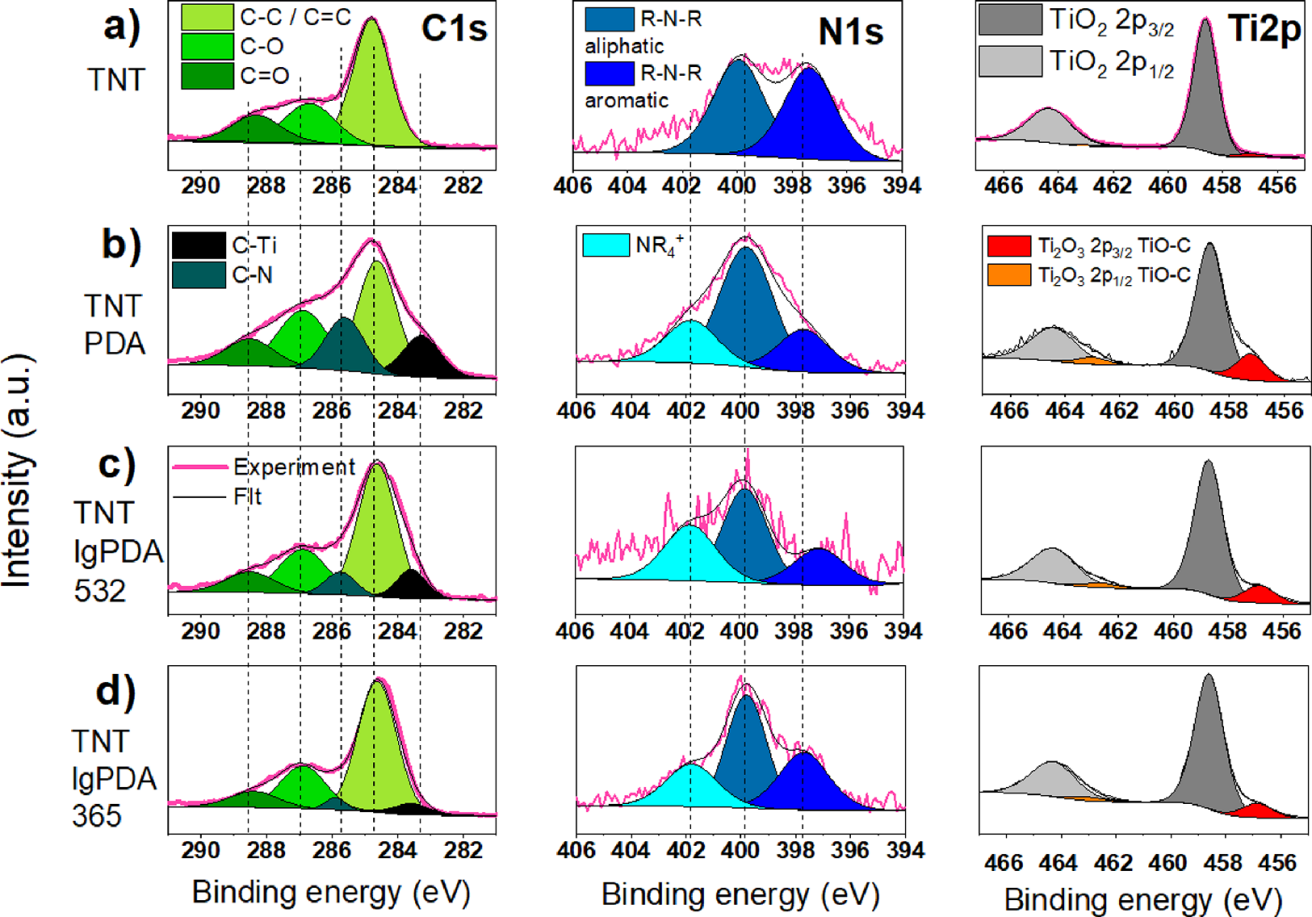
High-resolution XPS spectra in the carbon (C 1s), nitrogen (N 1s), and titanium (Ti 2p) regions: (a) pristine TNTs (titania nanotubes), (b) TNT_PDA (TNT covered with polydopamine), (c) TNT_lgPDA_532 (TNT_PDA graphitized using 532 nm laser), and (d) TNT_lgPDA_365 (TNT_PDA graphitized using 365 nm laser).

To investigate the changes in surface chemistry after each modification step, a series of high-resolution XPS measurements were performed (Figure 3 and Table 1). In general, besides the Ti 2p doublet, there are nonzero carbon and nitrogen signals even on the pristine TiO_2_ nanotubes (24 and 3%, respectively). They most probably originate from the electrolyte used during anodization and adventitious carbon layer formation under atmospheric exposure.

**Table 1:** Quantitative Data from the XPS Experiment Depicted in Figure 3

chemical group	TNT	TNT_PDA	TNT_PDA_532	TNT_PDA_365
C–C/C=C	14.55	25.01	20.62	30.43
C–O	5.88	16.36	8.41	11.6
C=O	3.94	7.63	4.12	4.78
C–Ti		9.15	3.55	2.44
C–N		11.66	2.72	1.45
**total C**	**24.37**	**69.81**	**39.42**	**50.7**
**total O**	**52.22**	**20.77**	**39.60**	**34.38**
R-N-R aliphatic	1.66	4.09	1.05	1.59
R-N-R aromatic	1.58	1.45	0.48	1.04
NR^4+^		1.46	0.74	0.79
**total N**	**3.24**	**7.00**	**2.27**	**3.42**
Ti 2p_3/2_ TiO_2_	19.47	2.02	16.38	10.32
Ti 2p_3/2_ Ti_2_O_3_	0.7	0.41	2.32	1.17
**total Ti**	**19.54**	**2.43**	**18.70**	**11.49**

Overall, after electropolymerization, the atomic carbon and nitrogen contents are increased to 70 and 7%, respectively, confirming the presence of the PDA, whereas the titanium content is reduced from 20 to 11%. Moreover, two new peaks emerge at the carbon spectrum at 283.3 and 285.7 eV associated with C–Ti and C–N bonds, respectively. The former can be interpreted as a noncovalent interaction of the PDA structural units with the surface layer of the TiO_2_ rather than the formation of titanium carbides. This statement has been elucidated in our previous work and supported by DFT calculations.^56^ The latter is a direct consequence of introducing PDA, which contains C–N bonds of different order, depending on the oxidation state of each unit. Such bonds are also reported on the nitrogen spectrum at 397.8, 399.9, and 401.8 eV associated with aromatic (imine) R-N-R groups, aliphatic R-N-R groups, and tetraalkylammonium (NR_4_^+^) groups, respectively.^56,70−72^ However, one should consider that noisy N 1s spectra of TNT IgPDA 532, originating from the low N concentration, affect deconvolution certainty. Additionally, another doublet appeared on the Ti spectrum corresponding to the TiO-C interaction parallel to the data on the C spectrum.

Laser modification of the PDA using 532 nm wavelength yields a reduction of the carbon and nitrogen contents to 40 and 2%, respectively. This outcome strongly indicates that a significant portion of those PDA constituents has been either evaporated or incorporated into deeper atomic layers of the material. However, the general structures of carbon, nitrogen, and titanium spectra are preserved. It is to be noted though that the proportion of the C–C bond content to other types of carbon bonds is increased and the maximum of the C–Ti peak is shifted to larger binding energies. The former suggests that more carbon atoms within the surface layer are bonded to other carbons and nothing else. The latter indicates stronger binding of the organic layer to the titania surface. Both changes could be interpreted as symptoms of graphitization. Over the nitrogen spectrum, a notable decrease of the aliphatic R-N-R group content with respect to others is observed, strongly suggesting a higher level of conjugation characteristic for the sp^2^ phase rather than sp^3^. Similar tendencies are observed in the case of the 365 nm modification, although the carbon and nitrogen contents did not plummet as much (to 51 and 3%, respectively).

Overall, these observations support the statement that graphitization occurred in the case of both the 532 and 365 nm laser exposures, and presumably, a smaller amount of the PDA evaporated in the 365 nm case.

### 3.5 ReaxFF Molecular Dynamics Studies of the Laser Graphitization Mechanism

There have been several mechanisms developed to explain the formation of nanocarbons from different organic precursors during thermal or laser treatment.

From the macroscopic thermodynamic perspective, thermal graphitization can be viewed as series of phase transition of the precursor and colling back to the stable graphitic phase (dissolution–precipitation).^33,73^ This process can be catalytically enhanced by the presence of metal, which is capable of forming a solid solution with a carbon source, effectively reducing temperatures of phase transitions. Intermediate carbide formation can also contribute to this catalytic effect.

From the nanoscopic perspective, particles of metal catalysts (e.g., Fe, Ni, Co) facilitate the formation of the planar carbon from the sp^3^-rich sources leading to the formation of graphite/graphene around particles. Then, “planarization” of the carbon propagates away from the catalyst. This can be clearly seen on the TEM images.^38,73^

In the case of lasers operating in the IR range, e.g., CO_2_ (10.6 μm), organic matter is initially converted to the amorphous carbon during the photothermal process followed by phase transition to graphite/graphene upon subsequent lasing.^47^ This mechanism seems to be general for both saturated and unsaturated precursors being π-conjugated or not. That is because the laser interacts with vibrational modes of C–C bonds that are abundant in any organics. Addition of metal catalysts prior to graphitization can also make this mechanism more feasible.^74^ In essence, the IR laser graphitization mechanism is very similar to the standard thermal (furnace) treatments.

On the other hand, the mechanism of UV or vis laser graphitization is based on photochemical conversion, where chemical bonds break directly as a result of optical excitations without the local temperature rising.^29^ In this case, the organic precursor must possess optical absorption bands (typically associated with a pi conjugated structure), and the laser power/fluence needs to cross a certain threshold. Details of this mechanism are the least known for any polymer and require further research combining experimental and theoretical approaches.

As an attempt to support the ideas of the PDA graphitization occurring in the current experiments, a series of molecular dynamics (MD) simulations of this process have been performed using reactive force field ReaxFF.^60^ The scheme of simulations was adapted from the work in other polymers prone to form laser-induced graphene/graphite.^62^

**Figure 4:**
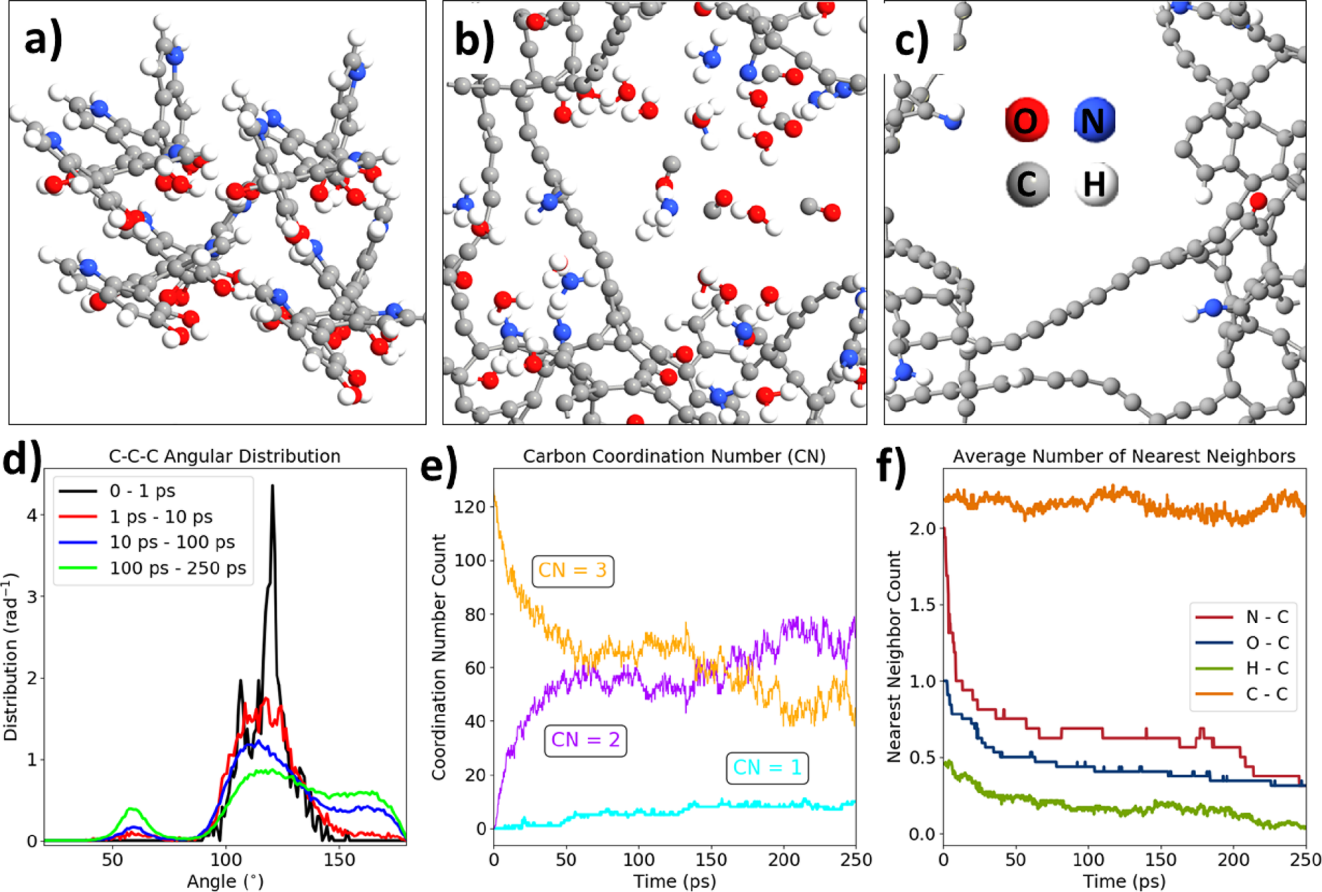
ReaxFF molecular dynamics of the early graphitization stage: (a) initial geometry of the system (4 DHI tetramers); (b, c) geometry of the whole system and the carbon backbone after 250 ps of the annealing at 3000 K; (d) angular distribution function for several periods of the trajectory; (e) evolution of the carbon coordination number; and (f) evolution of the carbon nearest neighbor number projected onto N, O, H, and C atoms (1.8 Å cutoff).

In general, an initial model of the PDA consisted of four linear tetramer geometry optimized in the periodic boundary conditions. The density of molecules was set to 0.6 g/cm^3^ to maintain similar conditions as in a previous work,^62^ which was equal to 0.8 g/cm^3^. A smaller density was taken to maintain sufficient free space for the flat graphene formation. During the first 250 ps of the NVT-MD, the initial stacked structure of tetramers begins to rupture with C–C, C–N, and C–O bonds breaking (Figure 4a,b). Then, the evolution of CO, H_2_, H_2_O, and NH_3_ gaseous products occurs. At the same time, the sp^2^ ringed carbon structures partially unfold and temporarily form sp chain-like entities (Figure 4c).

This initial loss of the ring structure can be seen on the angular distribution functions (Figure 4d) depicting the number of C–C–C bond angles per angle. Initially, most of the carbon atoms formed between 100 and 140° angles corresponding to five-membered and six-membered rings. As the trajectory progresses though, there are C–C–C fragments formed with 60 and 175° with the main 120° contribution plummeting. Second, the time evolution of the carbon coordination numbers (Figure 4e) shows a significant increase of the two-coordinated carbon in exchange with a decrease of the three-coordinated carbons. Thus, the shift from sp^2^ to sp during the initial phases of graphitization is justified. Moreover, there is a notable rise in the contribution of singly coordinated carbons corresponding to the evolution of CO gas. The gas evolution can also be tracked by the analysis of the nearest neighbor counts for C–N, C–O, and C–H bonds (Figure 4f). In general, the number of C–N, C–O, and C–H bonds rapidly declines throughout the first 100 ps, whereas the number of C–C bonds oscillates between values of 2.0 and 2.4. Thus, the gas evolution is restricted mostly to the time scale of 100 ps.

**Figure 5:**
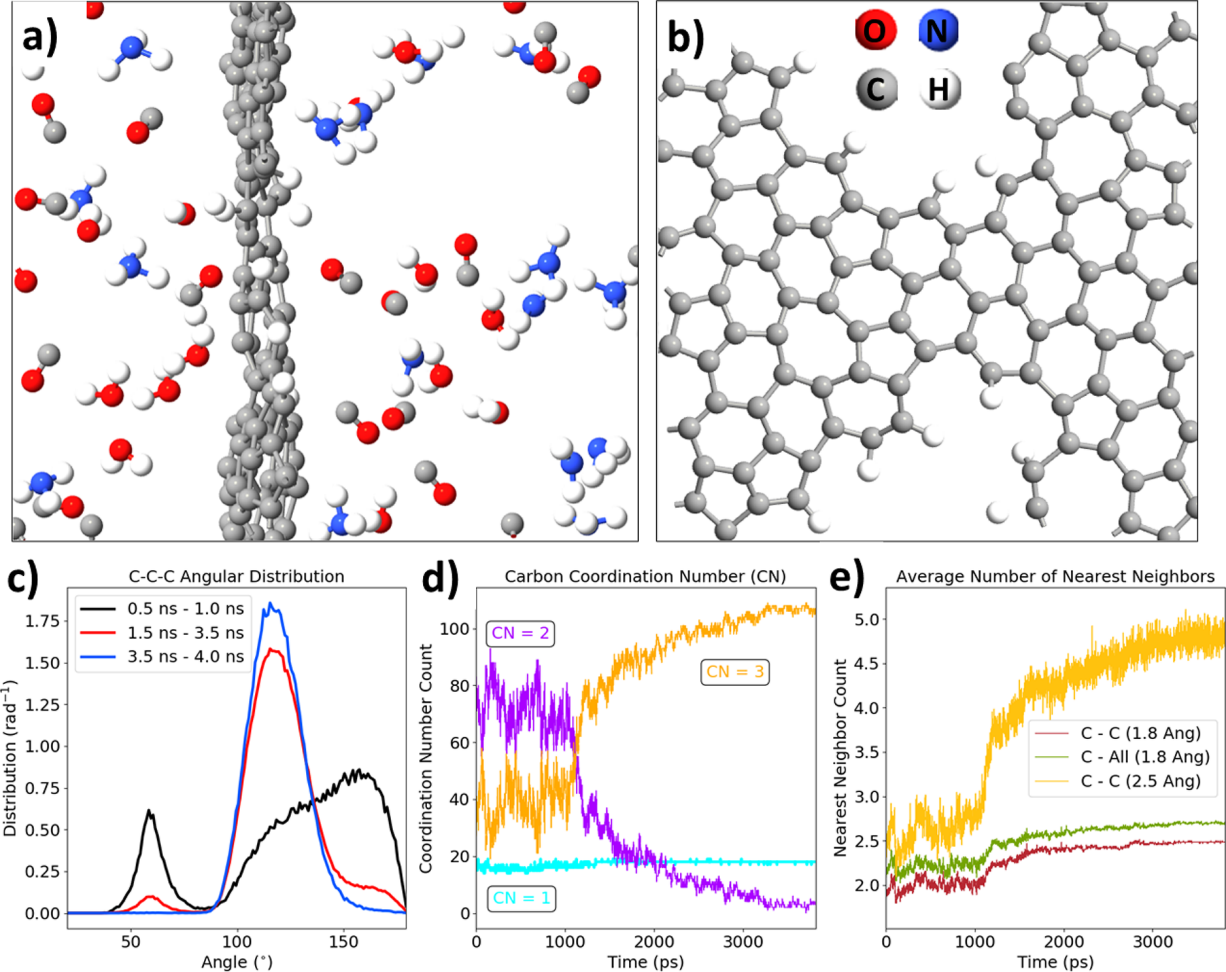
ReaxFF molecular dynamics of the late graphitization stage: (a) final geometry of the system after 4 ns of annealing at 3000 K; (b) top view of the resulting graphene plane; (c) angular distribution function for several periods of the trajectory; (d) evolution of the carbon coordination number; and (e) evolution of the carbon nearest neighbors calculated with different cutoffs (1.8 or 2.5 Å).

The latter part of the graphitization is mostly condensation of the unfolded structure into the sp^2^ graphene-like structure, which is achieved after 4 ns simulation in total (Figure 5a,b). The final lgPDA molecule is equivalent to a slightly twisted defect graphene with hydrogen terminations. The folding back process is captured on the angular distribution functions in Figure 5c. Most of the folding occurs between 1 and 3 ns, and after 3.5 ns, all carbon atoms not evolved to the gas phase constitute a single graphitic molecule, and all C–C–C angles are 120 ± 20°. The evolution of the carbon coordination number shows the opposite trend with respect to the initial part of the trajectory (compare Figure 5d with Figure 4e). An increase of the three-coordinated carbon numbers in exchange for two-coordinated ones corroborates the folding back process into the sp^2^ phase. The nearly constant value of the singly coordinated carbons confirms that the gas evolution does not occur after 1 ns of simulation. Finally, Figure 5e shows that there is a steep rise in the number of carbon neighbors being equal to 2.5 for the 1.8 Å cutoff and 5 for the 2.5 Å cutoff. Those values are greater with respect to those of the pristine PDA (ca. 2). Moreover, almost all neighbors of carbons are other carbons, indicating the graphitization process.

Considering the ReaxFF results, the experimental time of the pulse equal to 8 ns is sufficient for graphitization to occur. The temperature of the NVT thermostat seems not to play a significant role in determining either the final structure or the chemistry of the evolved gases, provided that the bonds are broken during the initial 250 ps. The threshold for this to occur is 2000 K for the applied force field and thermostat parameters (results not shown). Therefore, regardless of the actual initial temperature inside the material after the laser pulse, it is anticipated that the graphitization should occur for a wide range of lasing parameters including energy density, wavelength, and number of pulses.

Furthermore, the final structure of the computed lgPDA was withdrawn from the box and DFT optimized. Then, the band structure and density of states were calculated and compared to those of the pristine DHI tetramer before graphitization (Figure S14a,b). In general, the twisted structure of the hydrogenated graphene is preserved upon the DFT optimization with hydrogen atoms terminating some of the defected areas. The band structure does not exhibit Dirac cone characteristics for ideal graphite;^75^ the gap at the Gamma point is open, corroborating the defected structure (Figure S14c). However, there is still a nonzero density of electronic states at the Fermi level indicating that there are some occupied states in the Brillouin zone making the effective electronic band gap equal to zero, in contrast to the DHI tetramer (Figure S14d and e). In the case of zero effective band gap, photocurrent generation would be hindered because of the lack of exciton separation.^76^ Although a partially graphitized PDA structure with a nonzero band gap could act as an enhancer of the TiO_2_ absorption in the visible range, the fully graphitized one is expected to inhibit its photocurrent generation. On the other hand, zero band gap scenarios should facilitate electrochemical reactions, making the surface act as a metal compared to pure titania.

### 3.6 Influence of the Laser Fluence and Pulse Number on the Properties of the TNT_lgPDA

**Figure 6:**
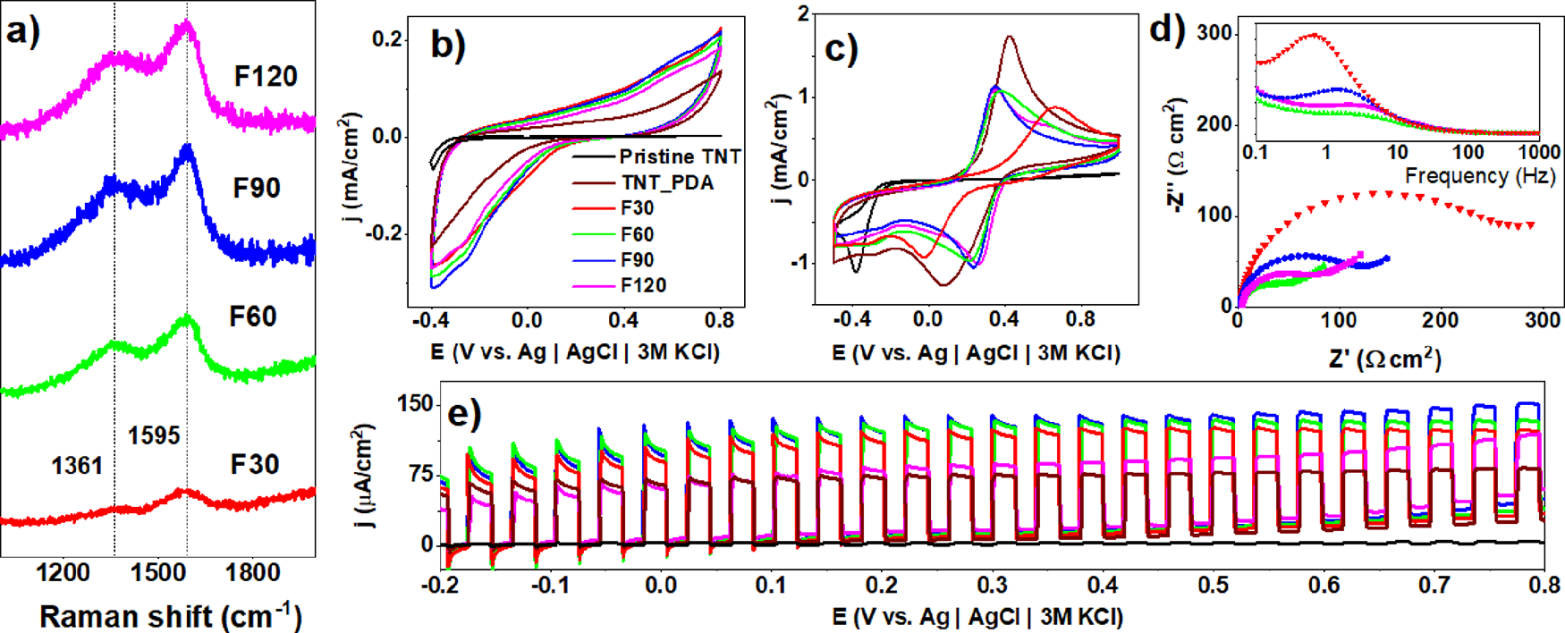
Optimization of laser fluence for TNT_lgPDA modified using a 532 nm wavelength. Effect of the laser fluence on (a) Raman spectra, (b) CV curves in the dark (0.5 M Na_2_SO_4_), (c) CV curves in the presence of ferrocyanides and ferricyanides (5 mM Fe(CN)_6_^3–^/5 mM Fe(CN)_6_^4–^), (d) corresponding EIS spectra, and (e) photocurrent–potential dependence in the visible part of the solar spectrum (0.5 M Na_2_SO_4_). F30–F120 values correspond to the range of laser fluences between 30 and 120 mJ/cm^2^.

Besides the laser wavelength, two other crucial parameters, the energy of the pulse (fluence) and the number of pulses, can strongly influence both the physical and chemical structure of the TNT_lgPDA material. The latter parameter was manipulated indirectly by changing the speed of the motorized table during the laser exposure; higher speeds are equivalent to a smaller number of pulses. Results in Figure 6 thoroughly describe the influence of those parameters on the Raman spectra and electrochemical and photoelectrochemical properties in the case of the 532 nm exposure.

In general, intensities of D and G bands increase with the fluence of the laser beam used for sample treatment (Figure 6a). Considering the rapid increase in both intensities at 60 mJ/cm^2^ with respect to 30 mJ/cm^2^, the former can be associated with the energy threshold required for graphitization. Regardless of this, all coatings slightly increase the capacitive background current with respect to the pristine PDA (Figure 6b).

Moreover, ferrocyanide redox currents are decreased, but the potential difference between them is shrunken (Figure 6c) except for the sample treated with 30 mJ/cm^2^ fluence. This decrease indicates that after graphitization using 532 nm wavelength, the reversibility of the surface reactions and thus the charge transfer rate has been enhanced. A similar trend is present in the EIS data (Figure 6d), with decreasing impedance magnitudes when the fluence is greater than 30 mJ/cm^2^. Compared with the nongraphitized PDA, the decrease is 2 orders of magnitude from 10 kΩ to 100 Ω (Figure S8). Moreover, characteristic time constants of the graphitized samples (2–5 Hz range) are smaller with respect to the nongraphitized PDA and 30 mJ/cm^2^ sample (0.8–0.9 Hz range). These trends strongly suggest that graphitized samples exhibit higher surface conductivity and charge transfer rates inherent to carbon-based surfaces. Therefore, it is anticipated that graphitization shifts the electronic structure of the PDA toward being more metallic.

Surprisingly though, vis-photocurrents are also increased as a consequence of graphitization (Figure 6e) across the wide window of polarization. Here, 30–90 mJ/cm^2^ fluences provide enhancement, whereas 120 mJ/cm^2^ does not. It suggests that too high of a fluence might cause too high of a degree of graphitization and metallic character. This in turn could plummet the electric field inside the space charge layer of the semiconductor and thus inhibit the capability of exciton separation and the photocurrent generation.^76^

**Figure 7:**
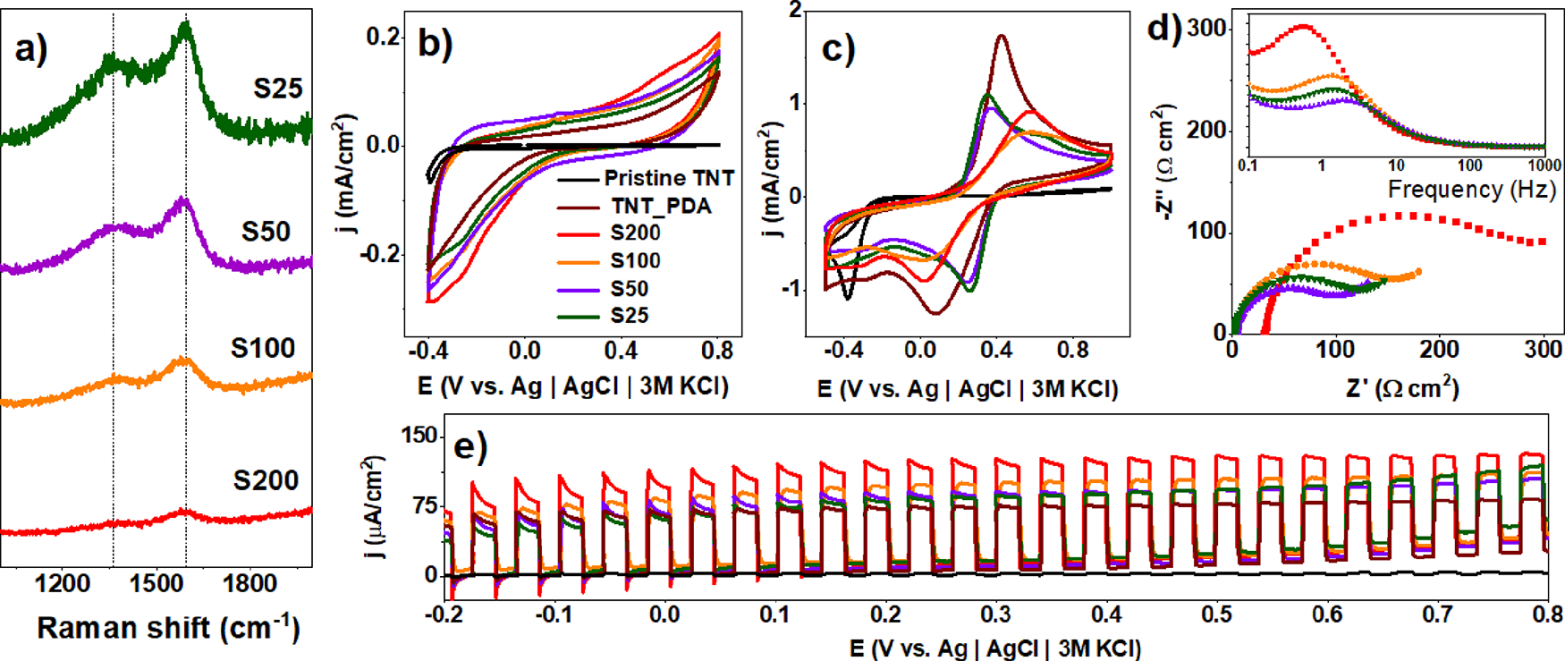
Optimization of the laser table speed for TNT_lgPDA modified using a 532 nm wavelength. Effect of the laser fluence on (a) Raman spectra, (b) CV curves in the dark (0.5 M Na_2_SO_4_), (c) CV curves in the presence of ferrocyanides and ferricyanides (5 mM Fe(CN)_6_^3–^/5 mM Fe(CN)_6_^4–^), (d) corresponding EIS spectra, and (e) photocurrent–potential dependence in the visible part of the solar spectrum (0.5 M Na_2_SO_4_). S25–S200 values correspond to the range of motorized table speed between 25 and 200 cm/h.

Exactly inverse trends can be captured with the speed of the table. Slower speeds corresponding to the higher number of pulses provide a gradual rise of both D and G Raman band intensities (Figure 7a). Similarly, capacitive background currents increase, (Figure 7b) and ferrocyanide redox peak current decreases (Figure 7c). Potential differences between peaks also decrease after graphitization provided the speed is less than 100 cm/h (S100), indicating a higher charge transfer rate and larger value of the characteristic time constant (Figure 7d). Additionally, Figure 7e shows that the vis-photocurrent enhancements are the most prominent only for samples modified with the highest laser table speed.

Such an outcome is in line with various literature showing the necessity of multiple lasing cycles for the efficient LIG-ation and enhanced electrical properties.^47,77^ This feature seems to be general and not limited for PDA.^29^

Analogous optimizations of laser parameters have been performed in the case of the 365 nm exposure, as depicted in Figure S9. Briefly, all symptoms of graphitization i.e., higher G band intensities and lower photocurrents, are obtained by higher fluence and slower table speed. However, electrochemical performance in the presence of ferrocyanides seems not to be altered significantly either by the fluence or the table speed. Presumably, the 365 nm laser interacting mostly with nanotubes enhances the reaction reversibility through charge transfer facilitation between lgPDA and nanotubes to a higher extent than modifications of the PDA itself. These properties are generally also reproduced when the laser graphitization is performed on the PDA deposited on the loosely spaced nanotubes (Figure S10) instead of adjacent ones described throughout the text.

### 3.7 Optimization of Lasing Parameters on TNT_lgPDA Photoelectrochemical Properties

**Figure 8:**
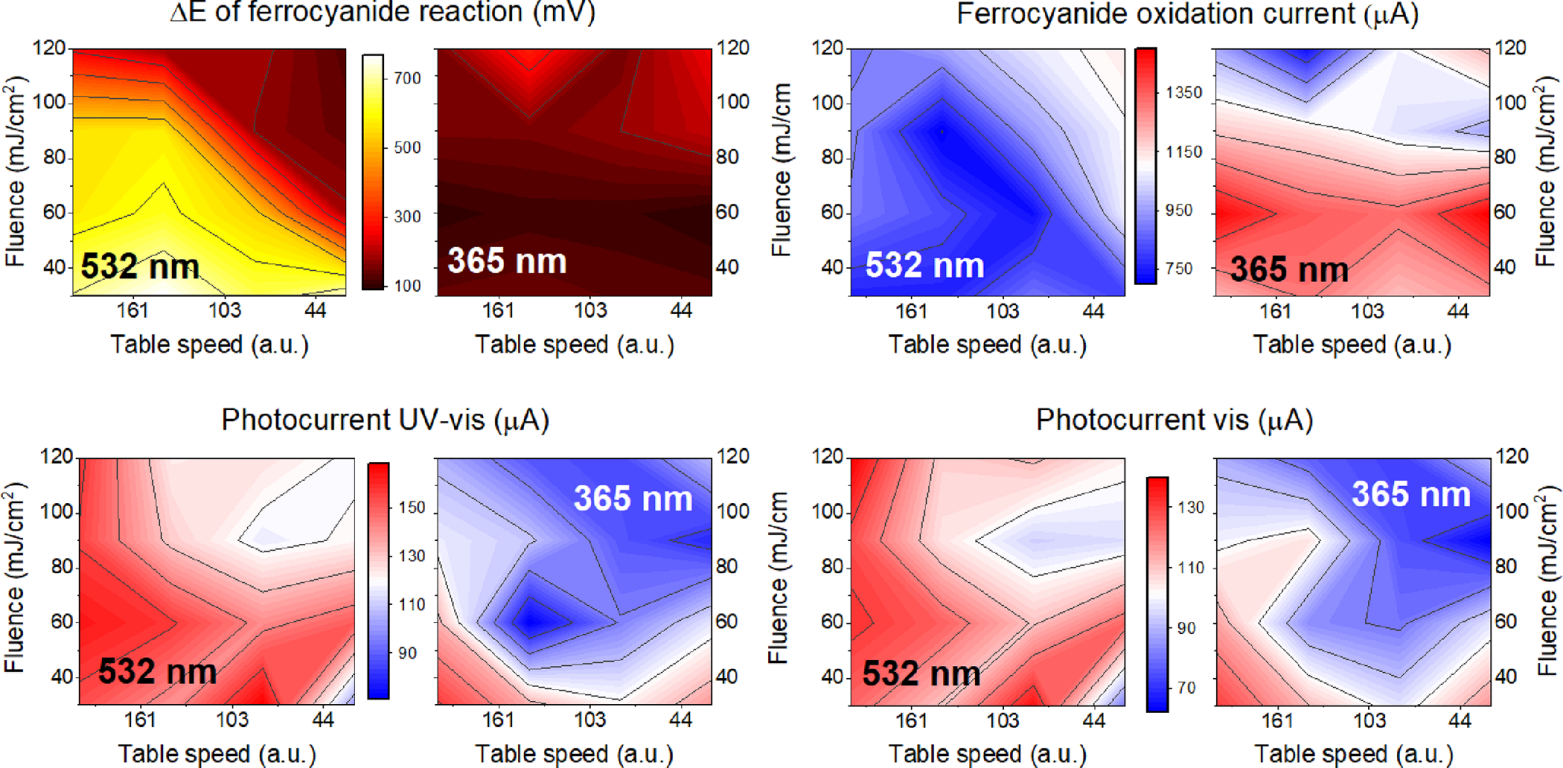
Contour maps illustrating the influence of laser wavelength, fluence, and number of pulses on various properties of the TNT_lgPDA including the (a) potential difference between ferrocyanide redox peaks; (b) currents at the oxidation maximum; and (c) photocurrents in the UV–vis and (d) vis parts of the solar spectrum. The right direction at each map points to a higher number of pulses, and the top direction points to a higher fluence. The left panes of each doublet correspond to the 532 nm, and the right panels correspond to the 365 nm modifications.

In the context of the ferrocyanide experiment, two parameters are crucial for estimating electrochemical activity: separation between redox peaks and the current value of the oxidation peaks. The former reflects the reversibility of the charge transfer reaction because the basal TiO_2_ n-type semiconductor is not expected to generate an oxidation peak due to the large band gap and deficiency of the density of states during anodic polarization. Those parameters alongside photocurrents are plotted as contour color maps in Figure 8 for 532 and 365 nm modifications with various laser fluences and numbers of pulses.

In general, the redox peak separation (Δ*E*) varies significantly with lasing parameters. The first observed trend is that the 365 nm modification results in overall higher electrochemical reversibility (Δ*E* being in the order between 90 and 180 mV) compared to the 532 nm modification (100–750 mV span). For the 532 nm modification, where the laser is believed to interact mostly with the PDA layer, higher fluence and number of pulses lead to a significant reduction of Δ*E* (Figure 8a). Heuristically, this outcome suggests that the higher degree of graphitization improves reversibility. On the other hand, there is less variability in terms of the fluence and pulse number within the 365 nm results. Presumably, the interaction of the laser with nanotubes leads to a higher graphitization degree through thermal effects and heat dissipation rather than photochemical changes. Moreover, the magnitude of the ferrocyanide oxidation peak is markedly greater for the 3ω modified samples (up to 1.4 mA compared to 0.8 mA for 532 nm on average, Figure 8b). Higher currents are observed when the material was irradiated with lesser fluences (30 and 60 mJ/cm^2^), presumably because higher fluences strongly distort the geometry of nanotubes beneath PDA (as reported in refs (78,79)), thus reducing electroactive surface area.

In terms of the photocurrents, the relationships are almost the opposite because the 532 nm modification results in higher photocurrent under both vis and UV–vis irradiations (Figure 8 and dc). In the case of both wavelengths, lesser fluence and a smaller number of pulses correspond to greater photocurrents. In other words, if laser modification is too intense, the photocurrent yield plummets. There is an inverse dependence that the laser modification leads to higher electrochemical activity, ultimately yielding lower photocurrents and vice versa. To estimate the photosensitization mechanism, samples chosen for further photoelectrochemical investigations are the ones with the highest photocurrents. The parameters are the following: 60 mJ/cm^2^ in the case of the 532 nm exposure and 30 mJ/cm^2^ in the case of the 365 nm exposure. In both cases, the fastest table speed was chosen, corresponding to the smallest number of pulses the sample is exposed to.

### 3.8 Wavelength-Resolved Photocurrents and Quantum Efficiencies of the TNT_lgPDA

**Figure 9:**
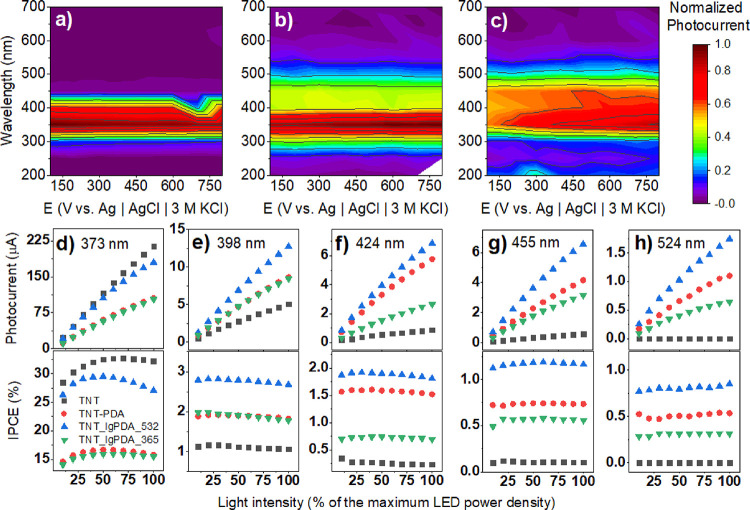
Photoelectrochemical properties of the TNT_lgPDA. Normalized photocurrent action maps for (a) pristine TNTs, (b) TNT_PDA, and (c) TNT_lgPDA modified with 532 nm, 60 mJ/cm^2^ laser. (d–h) Photocurrent and quantum efficiency dependencies of the studied samples on the monochromatic light intensity for several wavelengths: 373, 398, 424, 455, and 524 nm. One hundred percent of the LED power density is equal to 2.2 1.6, 1.3, 1.9, and 0.7 mW for all diodes, respectively.

To further elucidate the photoelectrochemical properties of the TNT_lgPDA heterostructure, a series of wavelength-resolved photocurrent measurements were performed (Figure 9). In Figure 6a, an action map of the normalized positive photocurrent shows that the main ability of the photocurrent generation occurs for the 300–420 nm range for the pristine nanotubes with the maximum at 350 nm. When the PDA is electropolymerized, this range is extended and covers the wavelengths between 260 and 540 nm with the minimum still at 350 nm. Finally, after the laser treatment, the photocurrent is generated for 300 to 550 nm, but the maximum is shifted to 400 nm, which is visible light. Moreover, whereas the pristine TNT and TNT_PDA do not exhibit significant variations of the photocurrent with the electrode polarization, it is notable in the case of the TNT_lgPDA (compare with Figures 6 and 7 and S9).

In general, there are several requirements for efficient photocurrent generation: photon absorption, exciton separation in the space-charge region, fast transport, and slow recombination. The stronger polarization required for the efficient separation suggests that the laser-graphitized electrode exhibits a weaker electric field in the space charge region, yet the photocurrents after modification are higher in magnitude. Therefore, intensity-modulated photocurrent–photovoltage spectroscopy IMPS/IMVS experiments have been performed to investigate the transport and recombination kinetics.

First, at the 373 nm illumination, the characteristic transport frequency is equal to 13–14 Hz for the samples modified with PDA and lgPDA compared to the 35 Hz of the pristine TNTs (Figure S11). There is also another characteristic frequency at ca. 700 Hz present only at the pristine TNTs. Those two frequencies presumably originate from two different electron transport pathways on the way to the terminal. This outcome suggests that modification with PDA—either graphitized or not—suppresses the transport component of the photocurrent generation requirements.

Moreover, in the regime of high frequencies over 1 kHz, the real part of the photocurrent becomes negative only for the modified samples. This “negative resistance” phenomenon might be associated with nonlinear effects of intensity-dependent transport or influence of localized surface states.^80,81^ This behavior is magnified even more in the experiment performed with 424 nm illumination when photocurrents are 1 order of magnitude less (Figure S12).

However, an advantageous attribute of graphitization can be captured in IMVS experiments (Figure S13) performed with 373 nm illumination. In particular, there is a recombination characteristic frequency at 0.15 Hz for the pristine TNT, which is reduced after PDA deposition to 0.04 Hz and even further after graphitization to the value of 0.02 Hz. The last value corresponds to the 4 s characteristic time of recombination. In conclusion, the anticipated mechanism of the photocurrent enhancement induced by the PDA and further graphitization is hindering the recombination.

Furthermore, photocurrent–light intensity (I–P) profiles are given for five different wavelengths (373, 398, 424, 455, and 524 nm) in Figure 9d–h with the corresponding values of the quantum efficiency (IPCE). I–P profiles are slightly nonlinear for all wavelengths in all cases for both pristine and modified electrodes. In the case of the 373 nm laser, photocurrents of the pristine TNT are comparable with the TNT_lgPDA treated with the 532 nm laser, and their IPCE value lies between 25 and 30%. It is to be noted that the IPCE varies quite significantly with the light intensity for this wavelength, most presumably due to nonlinear effects for the small intensities. However, at all the other wavelengths, photocurrents and quantum efficiencies exhibited by the TNT_lgPDA are several times higher compared to the pristine TNT. There is also a clear advantage of the 532 nm over the 365 nm exposure even for the optimized samples.

### 3.9 Photoelectrochemical Sensing of Serotonin

TNT_lgPDA composite can be considered as a member of the broad semiconductor–nanocarbon composite family. These materials are capable of photocurrent generation and have decent conductivity and charge transfer kinetics,^82−84^ although not as fast as, e.g., precious metals and alloys. In this context, the spectrum of possible applications of the TNT_lgPDA composite is spanned by several areas such as photoelectrochemical water splitting,^85^ removal of pollutants including antibiotics,^86^ and photoelectrochemical sensing (PEC).^87^ This subsection of results presents brief, proof-of-concept results showing the high propensity of the TNT_lgPDA toward PEC sensing of ultralow concentrations of serotonin in a neutral environment.

In general, serotonin was chosen because of the structural similarity to the PDA, which might facilitate amperometric detection.^16^ There are many important aspects in the field of neurotransmitter electrochemical sensing, such as spatial and temporal resolution, high selectivity, and long-term stability.^88^ However, for simplicity, the purpose here is to show how the graphitized PDA enables detection with a very low (below 1 nM) limit of detection without the support of any precious metals.

The mechanism of PEC detection is believed to be surface oxidation of the analyte by the photogenerated holes.^89,90^ In other words, the analyte acts as a hole scavenger.^84,91,92^ In this case, n-type semiconductors are used, and the photocurrent increase in the presence of analyte is also positive.^93,94^ However, there are n-type based PEC sensing materials, where the photocurrent decreases upon the addition of analyte.^90,95^ On the other hand, p-type semiconductors exhibit reverse trends and negative photocurrents.^96,97^

The sensing performance strongly depends on many factors such as the position of the Fermi level, tendency to Fermi level pinning, distribution of trap states, and experimental parameters.^83,92,98^ Nevertheless, the general intuition is that because of the excess energy of the photogenerated electrons, the interfacial charge transfer should be faster. Thus, the detection performance should in principle be better in the PEC experiments compared with the plain electrochemistry. Moreover, the signal-to-noise ratio is also typically smaller because the stimulus signal (light) is different from the collection signal (polarization).^82,94^

The pristine titania nanotubes do not show any affinity toward the serotonin oxidation either in dark conditions or after vis illumination in the desired concentration range (data not shown). The proposed TNT_lgPDA_365 composite in the dark conditions also does not exhibit any sign of serotonin oxidation (however, the background capacitive current is reduced upon serotonin adsorption, see Figure S15a).

**Figure 10:**
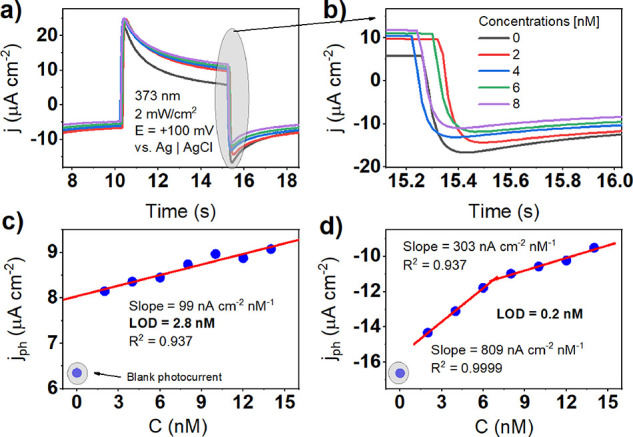
Photoelectrochemical chronoamperometric sensing of serotonin in 0.1× Tris (pH = 7.2) solution using TNT_lgPDA_365 nm: (a) shape of the photocurrent response; (b) magnification of the dark current just after switching off the light; (c) calibration curve plotted using photocurrent data; and (d) calibration curve plotted using dark current just after switching off the light. Polarization was equal to 0.1 V against Ag|AgCl |3 M KCl reference electrode. The light source was set to 373 nm LED with an intensity of 2 mW cm^2^.

On the other hand, when the light is applied, there is a notable increase in the photocurrent response upon the addition of serotonin in the nM concentration range (Figure 10). This happens both in the case of transient chopped light on/off as well as during continuous, potentiodynamic experiments (Figure S15). In the former case, photocurrent is calculated as the difference between the quasi-steady-state current during illumination (15 s at Figure 10a) and the current just before illumination (10 s at Figure 10a). The resulting calibration curve in Figure 10c shows a rapid increase of the photocurrent after addition of 2 nM compared to the blank 1× Tris solution and then a roughly linear increase until 16 nM. The calculated limit of detection (LOD) is equal to 2.8 nM; however, the rapid increase of the photocurrent after the first portion strongly suggests that its true value should be smaller. Moreover, the linearity is far from perfect.

Interestingly, when the dark current right after the light-off switch is taken as the main signal, the limit of detection is improved by 1 order of magnitude (200 pM), and two well-defined linear ranges emerge instead of one (Figure 10b,d). It shows, on the one hand, the complexity of the PEC sensing mechanism and, on the other hand, the very prominent behavior toward ultralow concentration detection. It is to be noted that a relatively small overpotential (+100 mV with respect to the ca. −100 mV of the OCP) is required for the effective PEC sensing and further anodic polarization does not improve the performance (results not shown). The sensing mechanism is even more complicated for higher concentrations of the analyte (Figure S15c,f). After exceeding ca. 16 nM, the photocurrent still increases, but the linearity is lost because of the current saturation of unknown origin. Moreover, the shape of the photocurrent response step changes markedly for some particular values of concentration.

According to the literature overview performed by the authors, there is no PEC sensor of serotonin published yet. However, there are some related works involving dopamine. Table 2 contains a comparison of the sensing performance against the plain electrochemical sensing of serotonin and the PEC sensing of dopamine. A more comprehensive discussion can be found in the review.^99^ The most prominent aspect of the TNT_lgPDA composite in comparison to other state-of-the-art materials is the lack of precious metals involved in the synthesis. Moreover, the fabrication protocol is simpler relative to other reports, and the material has only two constituents: titania nanotubes and graphitized PDA. Nevertheless, its limit of detection in a neutral (pH = 7.2) environment being equal to 200 pM is strongly competitive, and the sensitivity (defined as the slope of the linear range) is superior to all the other materials. The width of this range, related to the PEC sensing mechanisms, is to be improved though and requires more fundamental understanding of the photophysics of the detection. This is, however, in general not well understood in the literature.

**Table 2:** Comparison of Various Materials for the Electrochemical and Photoelectrochemical Detection of Neurotransmitters^a^

material	analyte	method and environment	limit of detection [nM]	linear ranges	sensitivity (slope) [nA cm^–2^ nM^^–^1^]	ref
CNTs-Cu2O-CuO@Pt	serotonin	CA (0.1 M PBS, pH = 7.4)	3	100 nM–2.5 mM	0.2	(100)
AuNPs@rGO/pTBA-Pd (C2H4N2S2)2/NF	serotonin	SWV (0.1 M PBS, pH = 7.4)	2.5	500 nM–200 μM	0.04	(101)
PEDOTNTs/rGO/AgNPs/GCE	serotonin	DPV (0.1 M PBS, pH = 8)	0.1	400 nM–44 μM	46	(102)
Ag/PPy/Cu2O/GCE	serotonin	DPV (0.1 M PBS, pH = 7.2)	12.4	10 nM–200 μM	0.08	(103)
Zn CNs-MWCNTs/H-Cu_2_O/CdTe QDs/GCE	dopamine	CA-PEC (0.1 M PBS, pH = 7.0)	0.3	1 nM–100 nM	unknown (logarithmic scaling)	(104)
BiPO4/BiOCl/g-C3N4	dopamine	CA-PEC (0.1 M PBS, pH = 7.0)	23	50 nM–10 μM	0.7	(105)
**TNT_lgPDA**	**serotonin**	**CA-PEC(0.1× Tris, pH = 7.2**)	**0.2**	2–68–16	**809 303**	**this work**

a CNTs = carbon nanotubes; rGO = reduced graphene oxide; pTBA = poly[2,2:5,2-terthiophene-3-(*p*-benzoic acid)]; PEDOT = poly(ethylene dioxytiophene); GCE = glassy carbon electrode; MWCTNs = multiwalled CNTs; QDs = quantum nanodots; SWV = square wave voltammetry; DPV = difference pulsed voltammetry, CA = chronoamperometry.

### 3.10 Discussion of Advantages and Disparities of the Laser Graphitization Protocol

Overall, the main advantages of laser graphitization over other graphitization methods, including high temperature treatment, are its speed, possibility of pattern formation, and relatively low energy expenditure. In principle, a single sample can be graphitized in less than a minute compared to thermal treatments requiring several hours (of course, the increment of graphitized surface area with lasing time depends on the spot size, which is adjustable). Adjustability of laser parameters enables tailoring of the morphology, porosity, and hydrophilicity of the obtained carbonized material.^30^ Moreover, in the case of PDA and other polymers (or even biomass components), temperatures required for carbonization are above 700 °C.^31^ Thus, a lot of energy is dissipated during furnace operation, in contrast to the laser treatment. Finally, the laser can enable formation of geometric patterns with the area graphitized selectively.

There are, however, disadvantages of the proposed method including the expensiveness of the laser device and optics as well as the necessity of lasing parameter fine-tuning. On the other hand, furnaces for thermal treatment can also be costly, especially because the presence of a vacuum or inert gas atmosphere is obligatory for PDA (and other polymers) carbonization. Despite the relatively high initial cost of the laser equipment, it is compensated over time due to large energy losses during thermal treatment. In the case of laser treatment, the presence of an inert gas or vacuum is not obligatory.^29,47^

In the opinion of the authors, the most problematic issue in the proposed method is the fine-tuning of the parameters. Although it was achieved in this study for titania nanotubes and PDA, each different pair of substrate and polymer would presumably require a different set of parameters for optimization toward electrochemical or photoelectrochemcial performance. This optimization for novel pairs might be associated with relatively high costs and in the case of some pairs not even achievable due to nonspecific surface interactions.

Interestingly, fluences used for PDA graphitization in this work are in the range of 30–120 mJ/cm^2^. On the other hand, typical values for other polymers are markedly higher. Athanasiou et al.^77^ used Nd:YAG 1064 nm laser to synthesize turbostratic graphene from biomass for supercapacitor applications. The fluence required for graphitization was more than 3 J/cm^2^ with the optimal value for electrochemical performance equal to 34 J/cm^2^. Fluence as large as 3 J/cm^2^ would cause full ablation of the PDA from the surface of TNTs. In other work, Duy et al. calculated the critical fluence of 5 J/cm^2^ for polyimide (PI).^106^ In the case of continuous lasers,^107^ the critical power was estimated to 2 W for PI and similarly to 1 W for the PDA in the seminal *Nature Communications* paper.^25^ Therefore, the possibility of PDA graphitization with significantly lower fluences is very promising. Of course, it raises the question of the physical origin of such an effect. On the one hand, it strongly suggests the catalytic effect of the TNTs toward PDA graphitization. On the other hand, it might be associated with the intrinsic propensity of the PDA toward graphitization. Further research is required to answer those questions and as a result develop applications.

## 4 Conclusions

In this work, laser-graphitized polydopamine is obtained on the surface of titania nanotubes via dopamine electropolymerization and subsequent pulsed laser treatment with 365 and 532 nm wavelengths. Partial graphitization is confirmed by Raman and XPS spectroscopies and supported by the water contact angle and electrochemical measurements. An increase in the PDA hardness irradiation was reproduced. Reactive molecular dynamics simulations have shown that graphitization is possible within the several nanosecond pulse time scale and CO, H_2_O, and NH_3_ gases evolve during the process. PDA tetramers are linked together into a single, slightly twisted carbon backbone with several defects and H terminations.

Whereas the 532 nm laser pulses interact mostly with PDA coating and graphitization through photochemical changes, the 365 nm pulses are absorbed by both PDA and the substrate nanotubes, leading to graphitization through both photochemical and thermal effects. As a result, the electrochemical activity is higher for 365 nm samples, but photocurrents are smaller. Although PDA already leads to the enhancement of the photocurrent and electrochemical activity of the titania nanotubes, after laser exposure, those are improved even more. Most of the photocurrent and quantum efficiency enhancement is observed in the visible light between 400 and 550 nm, and the boost is more prominent in the case of the 532 nm exposure. The anticipated mechanism of the photocurrent increase by laser modification is through slowing surface recombination rather than speeding up the charge transfer kinetics.

## Data Availability

The dataset underlying this work is openly available in Zenodo repository under the following persistent link: https://zenodo.org/records/10017804.

## Supporting Information Available

Materials and methods–supplementary: reagent synthesis of titania nanotubes (TNTs), X-ray photoelectron spectroscopy, diffuse reflectance UV–vis spectroscopy, Raman spectroscopy, and density functional theory calculation of the lgPDA electronic structure; results–supplementary: CV curves for dopamine electropolymerization on adjacent titania nanotubes, SEM images of the lgPDA on the surface of loosely spaced nanotubes, optimization of nanotube synthesis and PDA deposition parameters prior to graphitization, supplementary data on the physical properties of the TNT_lgPDA heterostructure, supplementary electrochemical and photoelectrochemical measurements for lgPDA deposited on adjacent and loosely spaced nanotubes, supplementary photoelectrochemical IMPS and IMVS measurements of the TNT_lgPDA system, and supplementary XPS quantitative data DFT calculations of the graphitized PDA electronic structure Supplementary serotonin sensing measurements (PDF)
