# Correction to “Laser-Induced Graphitization of Polydopamine on Titania Nanotubes”

**DOI:** 10.1021/acsami.4c15153

**Published:** 2024-09-18

**Authors:** Adrian Olejnik, Krzysztof Polaczek, Marek Szkodo, Alicja Stanisławska, Jacek Ryl, Katarzyna Siuzdak

In the original article, we investigated the process of laser graphitization of polydopamine deposited on titania nanotubes. For that purpose, we used two laser wavelengths, being second and third harmonics of the Nd:YAG laser as follows in the experimental section:

“Laser modification of samples aimed at PDA graphitization was performed using Nd:YAG (QSmart 850 Quantel) pulsed nanosecond laser using second (532 nm) and third (365 nm) harmonics.”

However, there is a typo throughout the paper because the third harmonic of the Nd:YAG (1064 nm) is equal to 354.66 nm (355 nm), not 365 nm. Therefore, all related abbreviations in the text and figures such as *“TNT_lgPDA_365”* should be *“TNT_lgPDA_355”.* We apologize for this mistake, which could be confusing for readers.

