# Supplementary material for: Laser-Induced Graphitization of Polydopamine on Titania Nanotubes
Source: ACS Appl Mater Interfaces. 2023 Nov 2;15(45):52921–38. doi: 10.1021/acsami.3c11580 (PMC10658452; doi:10.1021/acsami.3c11580)
Supplement: Supplementary file 1 — am3c11580_si_001.pdf [file am3c11580_si_001.pdf]

Supporting Information:
Laser-induced graphitization of polydopamine on titania nanotubes

Adrian Olejnik*[1,2], Krzysztof Polaczek[2,3], Marek Szkodo[4], Alicja Stanisławska[4], Jacek Ryl[5], Katarzyna Siuzdak[2]

[1]*Department of Metrology and Optoelectronics, Faculty of Electronics, Telecommunications and Informatics, Gdańsk University of Technology, Narutowicza 11/12 St., 80-233 Gdańsk, Poland Narutowicza 11/12 St., 80-233 Gdańsk, Poland*

[2]*Centre for Plasma and Laser Engineering, The Szewalski Institute of Fluid-Flow Machinery, Polish Academy of Sciences, Fiszera 14 St., 80-231 Gdańsk, Poland*

[3] *Department of Biomedical Chemistry, Faculty of Chemistry University of Gdansk, Wita Stwosza 63 St, 80-308 Gdańsk, Poland*

[4]Institute of Manufacturing and Materials Technology, Faculty of Mechanical Engineering and Ship Technology, Gdańsk University of Technology, Narutowicza 11/12 St., 80-233 Gdańsk, Poland

[5]Institute of Nanotechnology and Materials Engineering and Advanced Materials Center, Gdańsk University of Technology, Narutowicza 11/12, 80-233 Gdańsk, Poland

***Corresponding author: aolejnik@imp.gda.pl, Gdańsk University of Technology, 11/12 Narutowicza St. Gdańsk, Poland**

Materials and methods - supplementary

Reagents

10 X Tris Buffer was obtained from Santa Cruz Biotechnology and diluted 10 times prior to the measurements, dopamine hydrochloride was purchased from Sigma-Aldrich and the deionized water used in the experiment was provided by a Hydrolab HLP-5p system. Titanium plate (99.7% purity) was obtained from Strem Chemicals. Na_2SO_4, acetone, ethanol, diethylene glycol, HCl, NaOH, $K_3[Fe(CN)_6]$, $K_4[Fe(CN)_6]$, and NH_4F were provided by Chempur.

Synthesis of titania nanotubes (TNTs)

The protocols for titania nanotube synthesis were reported previously elsewhere.[27,28] Briefly, prior to anodization, the titanium plate was ultrasonically cleaned in acetone, ethanol and subsequently water. Single-step anodization of the titanium plate was performed in an electrolyte containing diethylene glycol / water / HF (69 / 5 / 1 vol. %) and 9.4 mM NH_4F. The anodization voltage was 30 V or 40 V (as indicated in the text), the polarization time was 2 h and the temperature was 40 °C. After anodization, the TNTs were rinsed with ethanol and dried in an air stream. Finally, the plates were calcined in a tube furnace (Nabertherm) at 450 °C for 2 hours.

X-Ray Photoelectron Spectroscopy

X-Ray Photoelectron Spectroscopy (XPS) studies were carried out on an Escalab 250Xi multispectroscope (ThermoFisher Scientific) operating with an AlKα X-ray source. The spot size was 650 µm and the pass energy through the hemispherical analyzer was 20 eV. Throughout the measurement, the samples were flooded with low-energy electrons and low-energy Ar^+ ions to assure charge compensation, with a final peak calibration at adventitious carbon C 1s (284.8 eV). The obtained spectra were analyzed and deconvoluted using the Avantage v5.9921 software (ThermoFisher Scientific).

Diffuse reflectance UV-vis spectroscopy

Diffuse reflectance spectra were collected with a PerkinElmer Lambda 35 (PerkinElmer, Waltham, MA, USA) dual-beam spectrophotometer in the range of 200–1000 nm with a scanning speed of 60 nm/min.

The Kubelka-Munk functions F(R) were calculated on the reflectance R data according to the Equation 2[1]:

$$F(R) = \frac{(1-R)^2}{2R} \quad (S1)$$

Raman spectroscopy

Raman spectroscopy measurements were conducted using a confocal micro-Raman spectrometer (InVia, Renishaw, Wotton-under-Edge, Gloucestershire, UK) with an argon-ion laser source emitting at 514 nm and operating with a power of 10 mW. Raw Raman data were normalized with respect to the largest anatase band (A band) and the fluorescence was subtracted to highlight the G/D band ratios. The deconvolution process involved fitting two straight lines so that the fluorescence background signal beneath the D and G bands is reduced to zero.

Density functional theory calculation of the lgPDA electronic structure

Density functional theory (DFT) computations were performed using the Perdew–Burke–Ernzerhof (PBE) functional within the generalized gradient approximation (GGA) implemented in the ATK package[2]. The Linear Combination of Atomic Orbitals (LCAO)[3] method with medium ATK basis set and Fritz-Haber Institute (FHI) pseudopotentials were applied.

The lgPDA structure was taken from the final frame of the ReaxFF simulation and all the gases were removed. Then, the resulting lgPDA sheet was DFT optimized with relaxed parameters and symmetry of the unit cell with 0.05 eV/Ang threshold.

Density of states and bandstructures were calculated with a 7×7×1 A^3 k-point mesh and a density mesh cutoff of 125 Ha. The DOS spectra of isolated PDA tetramer was computed for their corresponding optimized structures with multipole boundary conditions (single k-point).

Results - supplementary

CV curves for dopamine electropolymerization on adjacent titania nanotubes

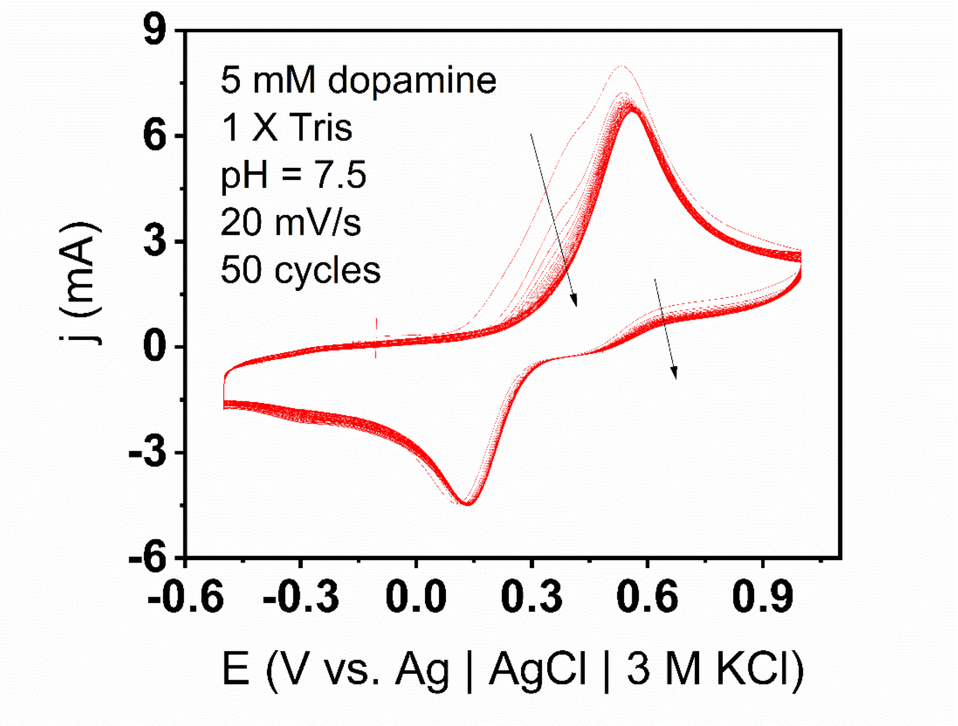

Figure S1. 50 cycles of cyclic voltammetry with 20 mV/s scan speed registered during dopamine electropolymerization in the Ar-purged solution containing 5 mM dopamine, 1 X tris and 0.5 M Na_2SO_4. Arrows indicate the direction of current changes during cycling.

SEM images of the lgPDA on the surface of loosely-spaced nanotubes

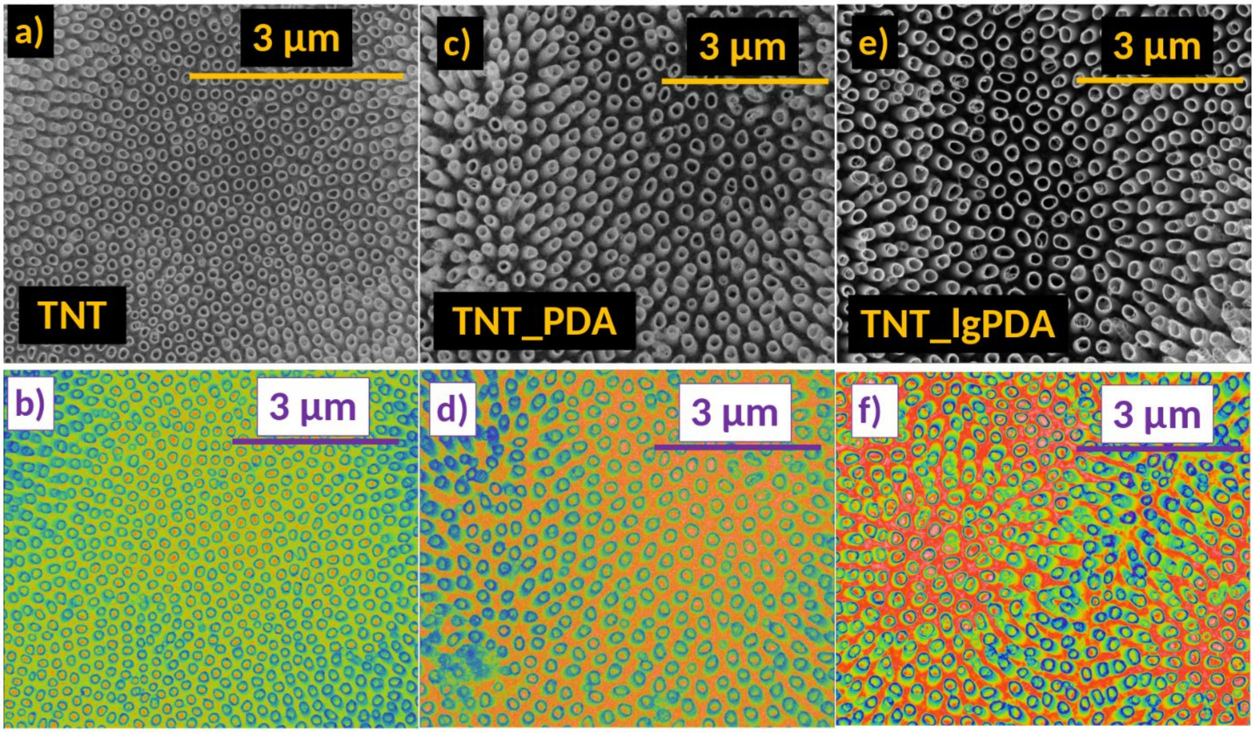

Figure S2. SEM images of the modified loosely spaced nanotubes; a-b) images of the pristine TNTs; c-d) images of the TNT_PDA before graphitization; e-f) images of the TNT_lgPDA with 532 nm exposure. Two contrasts of the same images are given for clearer representation.

Optimization of nanotubes synthesis and PDA deposition parameters prior graphitization

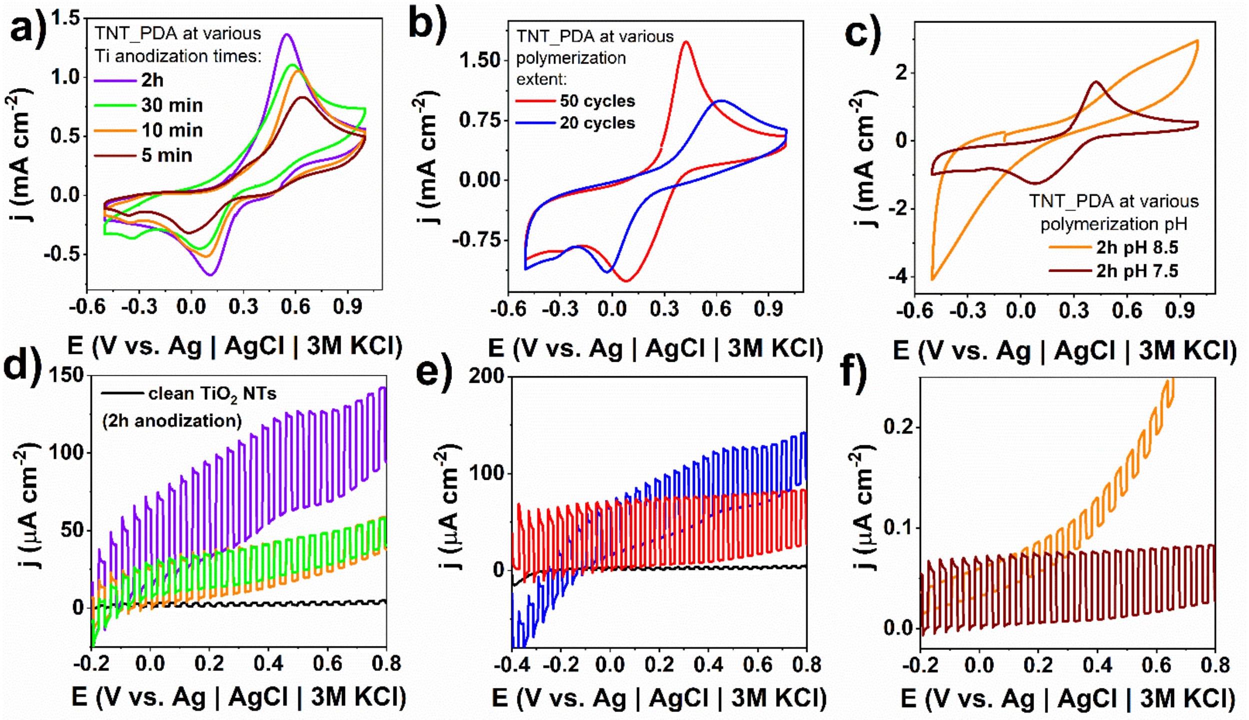

Figure S3. Optimization of the anodization and electropolymerization parameters prior graphitization; a) CVs of PDA electropolymerization given for nanotubes synthesized with varying anodization time – the first cycle of 50 is shown; b) CVs in ferrocyanide solution registered after PDA electropolymerization with varying number of cycles at pH = 7.5; c) CVs in ferrocyanide solution registered after 50 cycles of PDA electropolymerization with varying pH; d-f) visible photocurrent density – potential dependencies of TNT_PDA depending on the anodization time (d), number of PDA electropolymerization cycles (e) and electropolymerization pH (f). The b), c), e) and f) curves are presented for TNT_PDA samples with nanotubes synthesized during 2h anodization. Electrolyte for ferrocyanide experiments contained 5 mM $Fe(CN)_6{}^{3-}$ / 5 mM $Fe(CN)_6{}^{4-}$). Supporting electrolyte was 0.5 M Na_2SO_4 for every measurement.

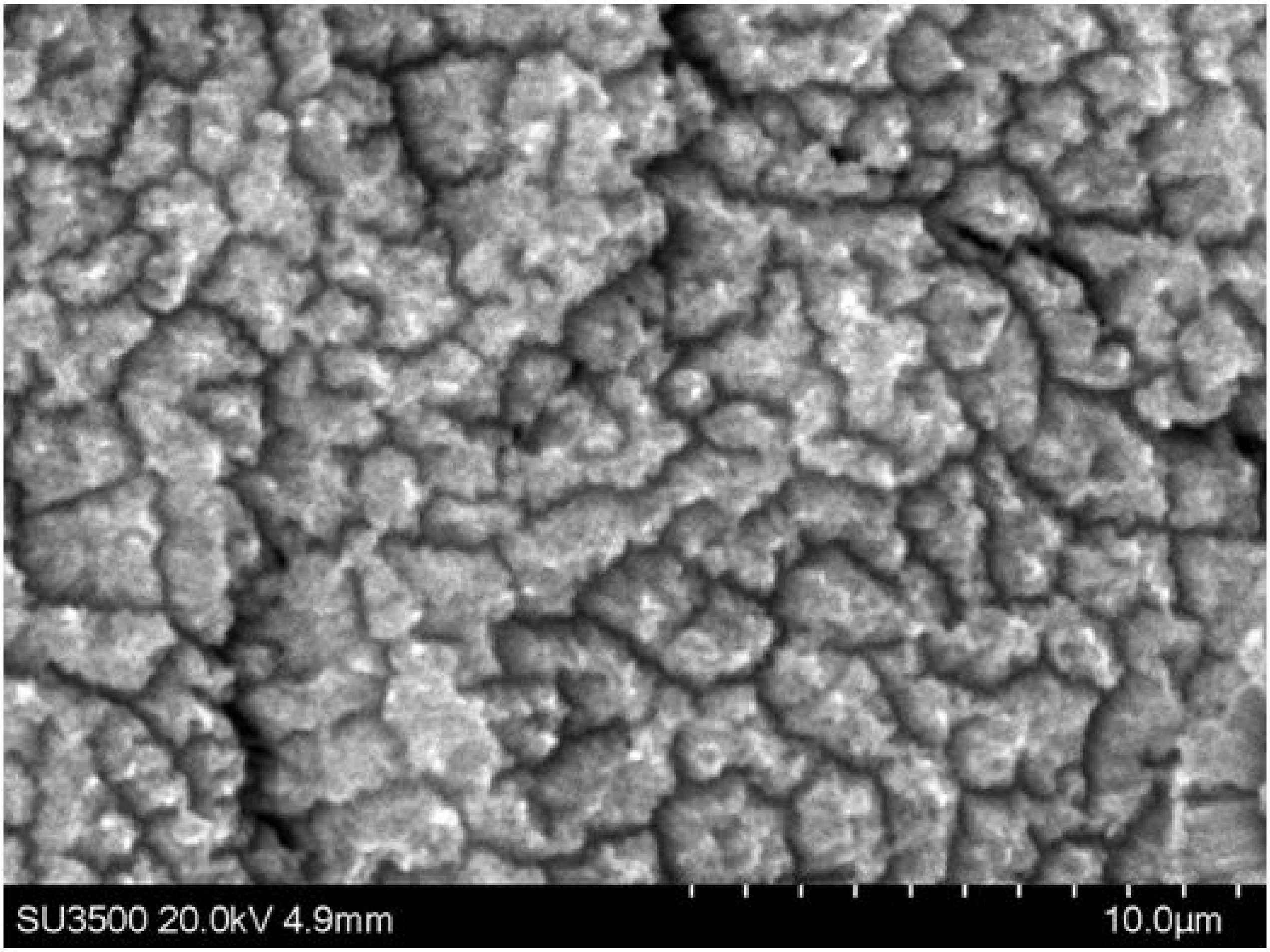

Figure S4. SEM image of the TNT_PDA structure based on the adjacent nanotubes fabricated during 3-hour anodization. Note the nanotubes morphology is lost after PDA deposition.

Supplementary data on the physical properties of the TNT_lgPDA heterostructure

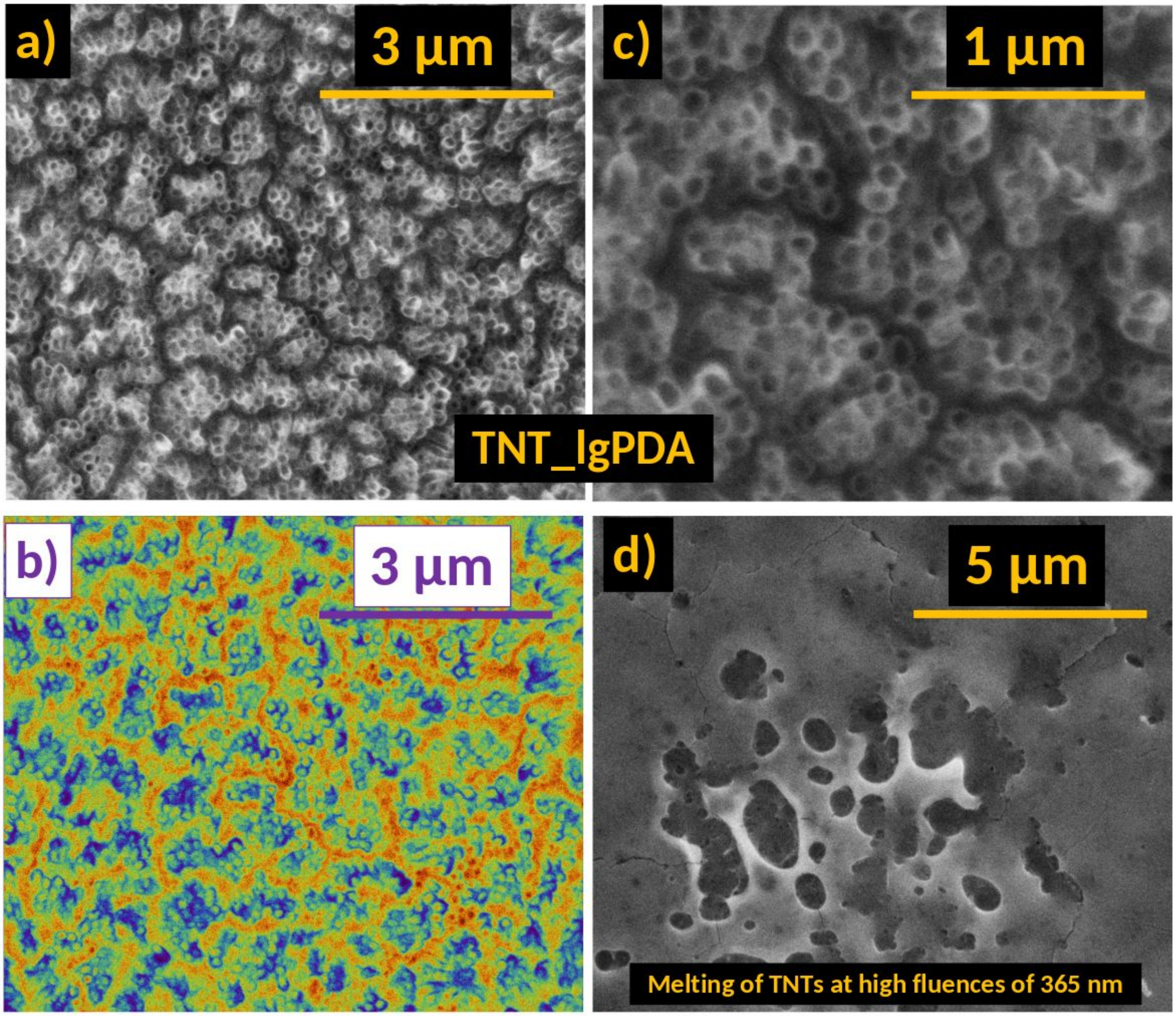

Figure S5. SEM images of the laser-graphitized adjacent nanotubes; a-b) images of the same TNT_lgPDA sample in two contrasts, c) magnification of the previous image, d) example of nanotubes melting, occurring only at higher fluences (90 and 120 mJ/cm^2) of the 365 nm laser exposure.

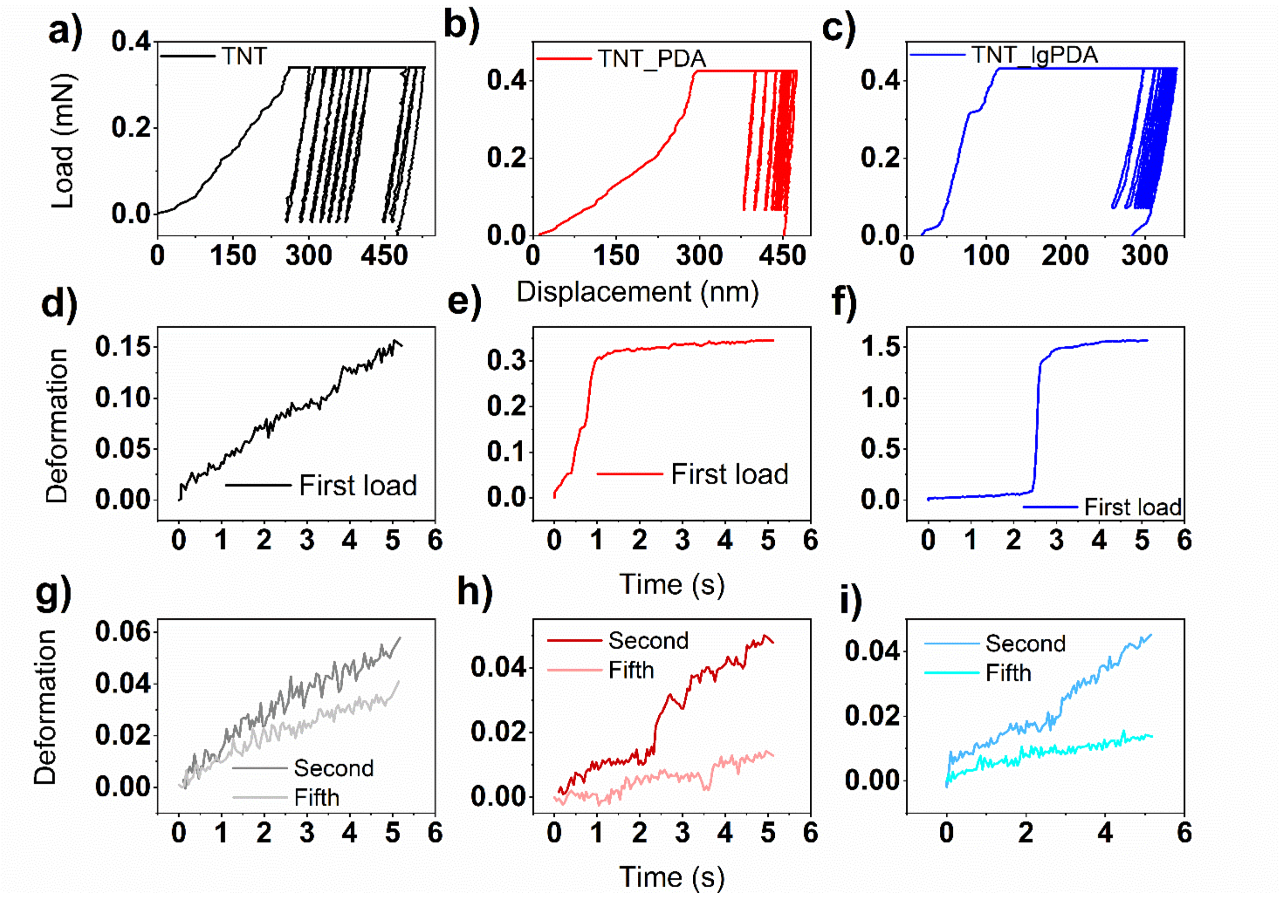

Figure S6. Details of the multiload nanoindentation experiments for pristine TNTs, TNT_PDA and TNT_lgPDA samples; a-c) load-displacement curves of the multiload procedure; d-f) deformation-time curves of the first indent; g-i) deformation-time curves of the second and fifth indents used for calculation of mechanical properties presented in the main text.

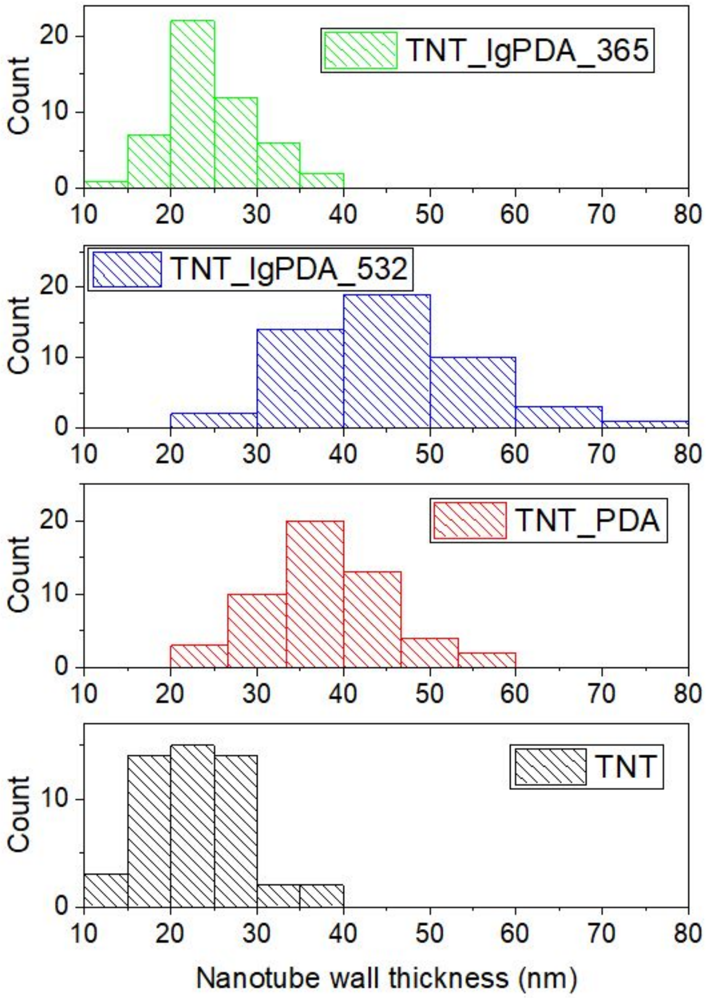

Figure S7. Statistics of the nanotube wall thickness of TNT, TNT_PDA, TNT_lgPDA samples calculated from the SEM images of the loosely spaced nanotubes in Figure S2 used to estimate the coating thickness given in Figure 2 in the main text.

Supplementary electrochemical and photoelectrochemical measurements for lgPDA deposited on adjacent and loosely-spaced nanotubes.

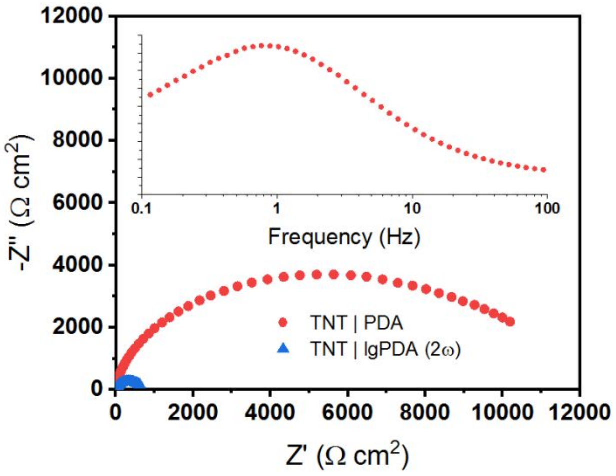

Figure S8. Comparison of EIS spectra for TNT_PDA and lgTNT_PDA.

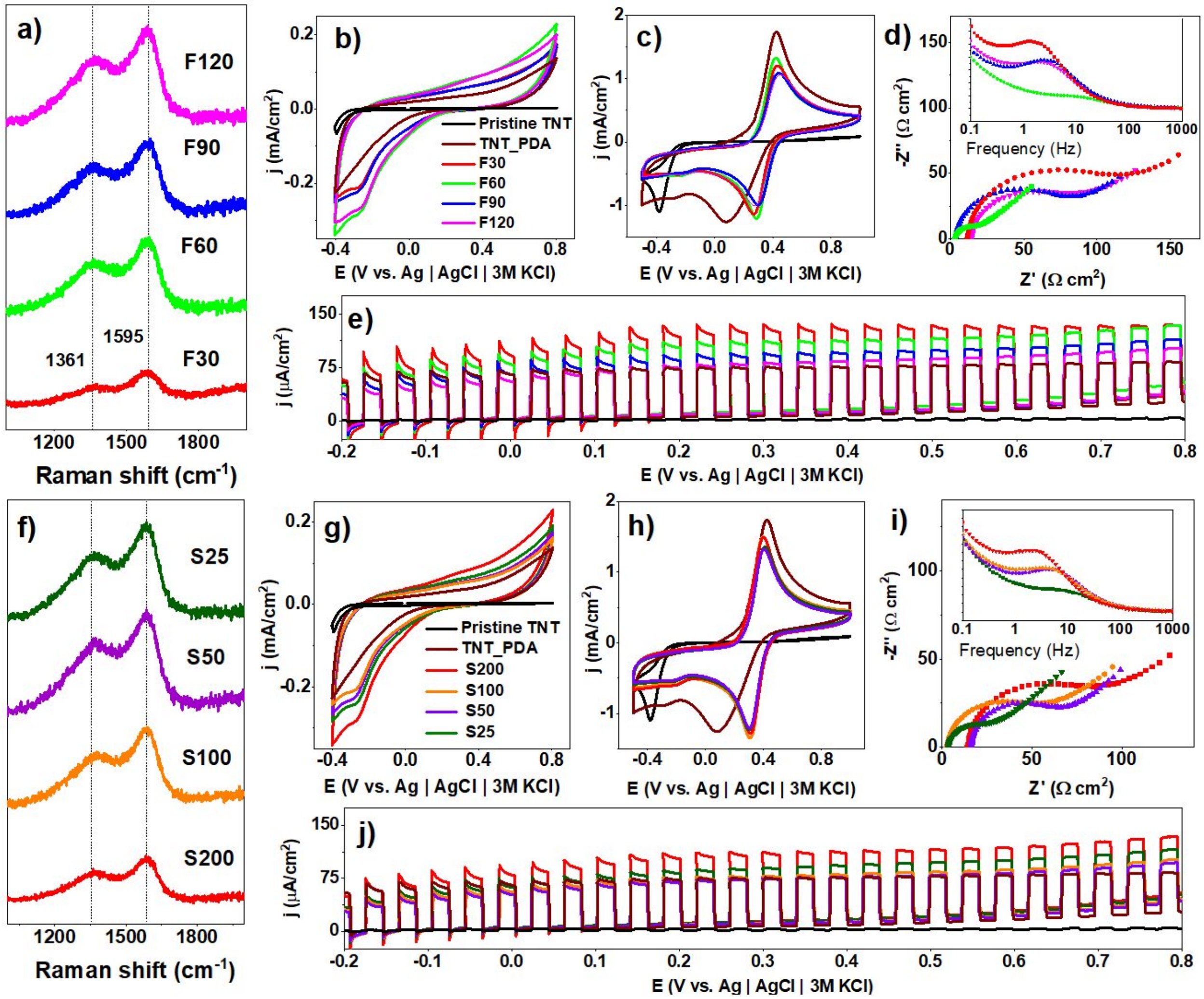

Figure S9. Optimization of laser parameters for TNT_lgPDA modified using 365 nm wavelength. Top pane illustrates the effect of the laser fluence on a) Raman spectra, b) CV curves in dark (0.5 M Na_2SO_4), c) CV curves in presence of ferrocyanides (5 mM $Fe(CN)_6{}^{3-}$ / 5 mM $Fe(CN)_6{}^{4-}$), d) corresponding EIS spectra, d) photocurrent – potential dependence in the visible part of the solar spectrum (0.5 M Na_2SO_4). F30-F120 values correspond to the range of laser fluences between 30 mJ/cm^2 to 120 mJ/cm^2. Bottom panel illustrates the effect of the number of the laser movement speed (inverse of the number of pulses) on f) Raman spectra, g) CV curves in dark, h) CV curves in presence of ferrocyanides, i) corresponding EIS spectra, j) photocurrent – potential dependence in the visible part of the solar spectrum. S25-S200 values correspond to the range of motorized table speed between 25 mm/min to 200 mm/min.

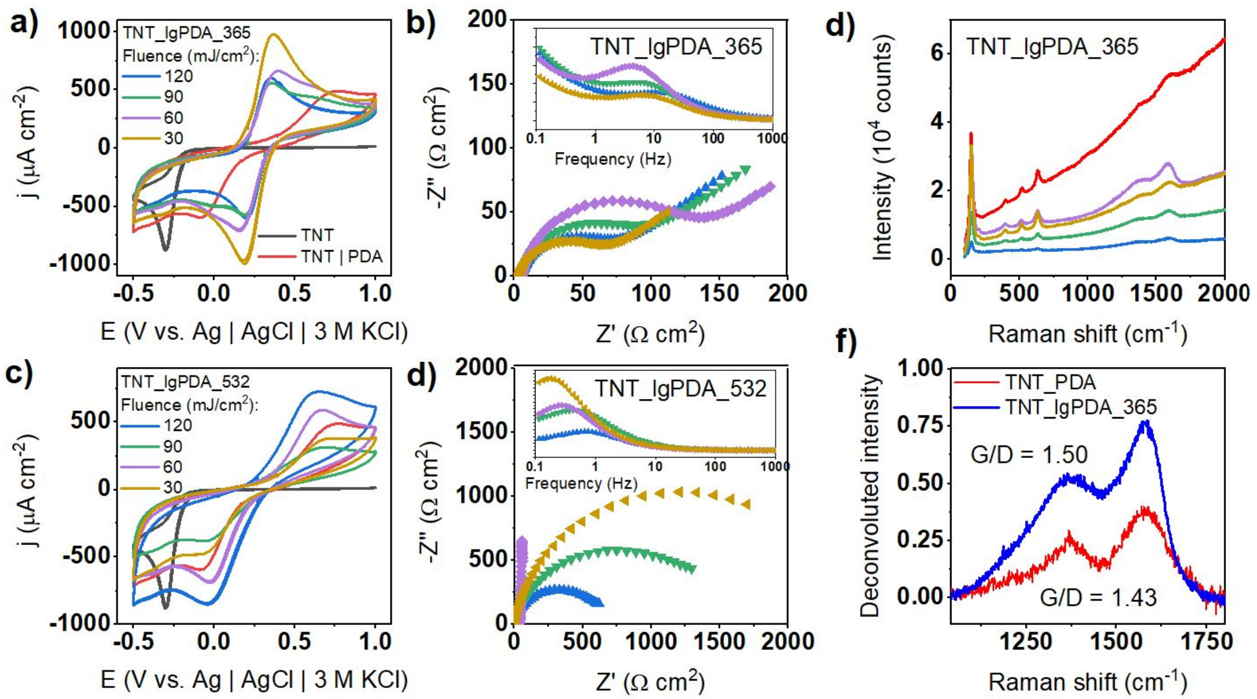

Figure S10. Electrochemical properties and Raman spectra of the TNT_lgPDA structures based on loosely spaced nanotubes; a-b) CV curves and EIS spectra obtained for different fluences of the 532 nm modification; c-d) CV curves and EIS spectra obtained for different fluences of the 365 nm modification; e) Raw Raman data for the 365 nm modification with different fluences; f) comparison of the Raman spectra obtained for the graphitized and pristine PDA after fluorescence subtraction.

Supplementary photoelectrochemical IMPS and IMVS measurements of the TNT_lgPDA system

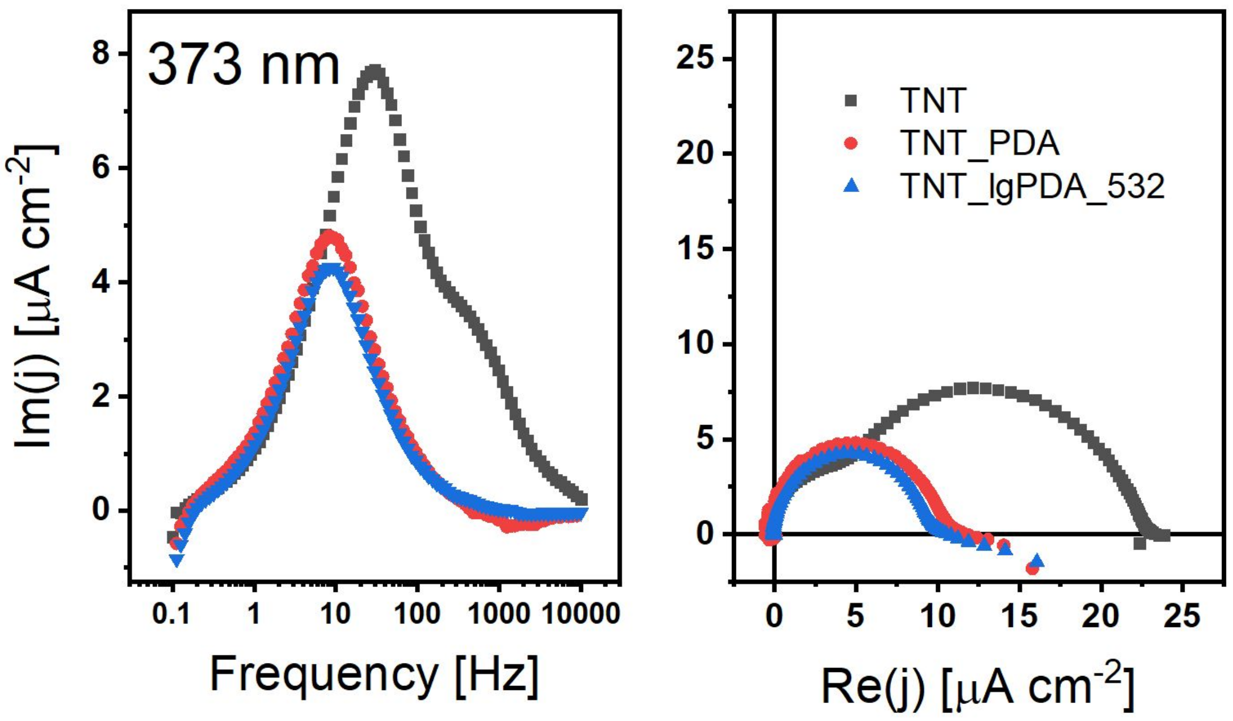

Figure S11. IMPS Bode and Nyquist spectra of the pristine and modified TNTs recorded during +500 mV polarization and AC/DC light intensity equal to 0.2 mW / 1 mW at 373 nm wavelength.

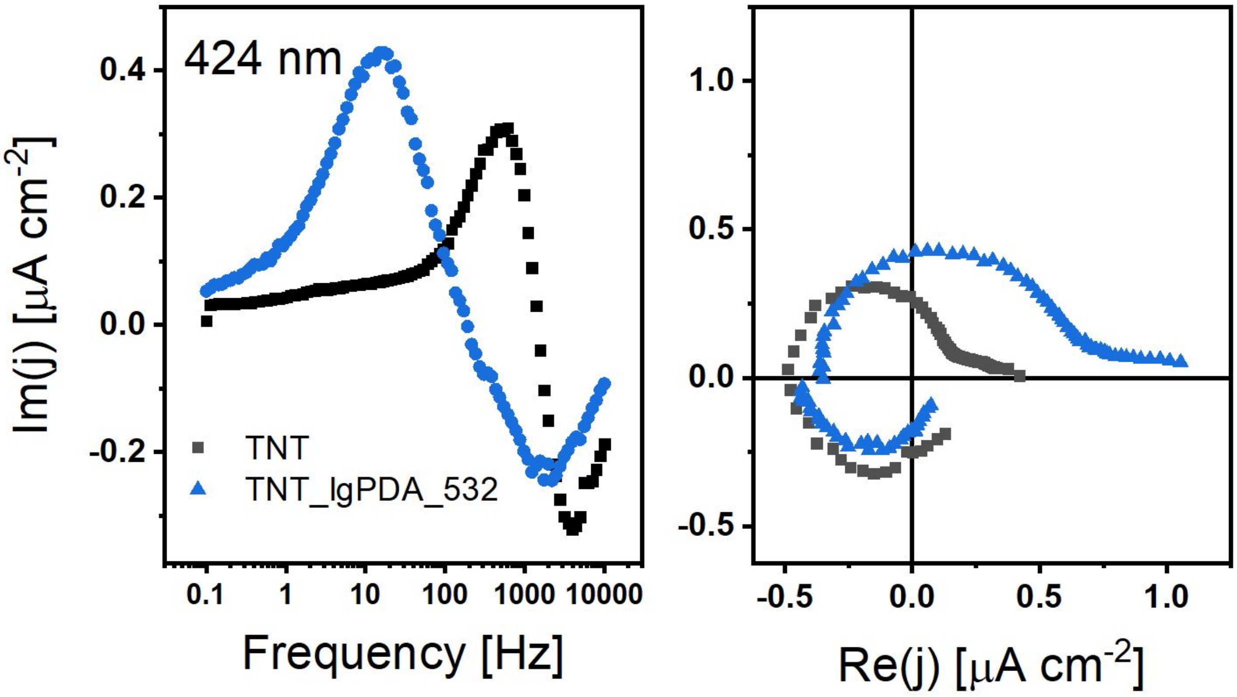

Figure S12. IMPS Bode and Nyquist spectra of the pristine and modified TNTs recorded during +500 mV polarization and AC/DC light intensity equal to 0.8 mW / 1 mW at 424 nm wavelength.

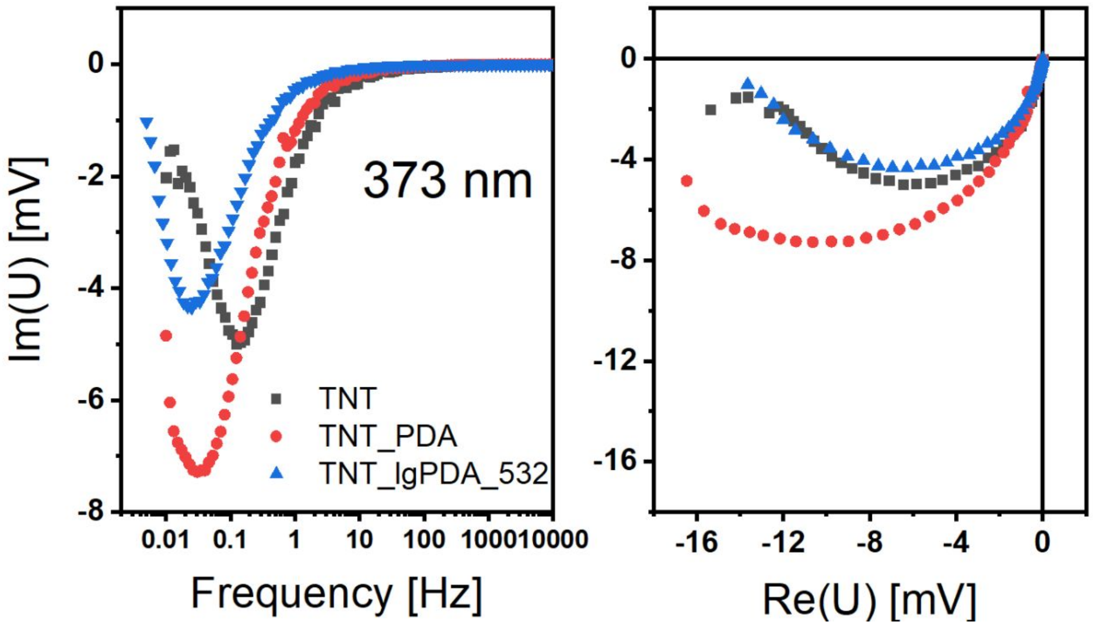

Figure S13. IMVS Bode and Nyquist spectra of the pristine and modified TNTs recorded in open circuit and AC/DC light intensity equal to 0.2 mW / 1 mW at 373 nm wavelength.

Supplementary XPS quantitative data

Table S1. Quantitative data from the oxygen area O1s of the XPS spectra.

Chemical group	TNT	TNT_PDA	TNT_PDA_532	TNT_PDA_365
O1s TiO2	34.81	5.3	22.22	20.02
O1s C=O	12.67	5.31	8.41	7.62
O1s Ti-OH	3.4	4.05	5.81	3.96
O1s CO	1.34	4.05	2.55	2.16
O1s ???	-	2.06	0.61	0.62

DFT calculations of the graphitized PDA electronic structure

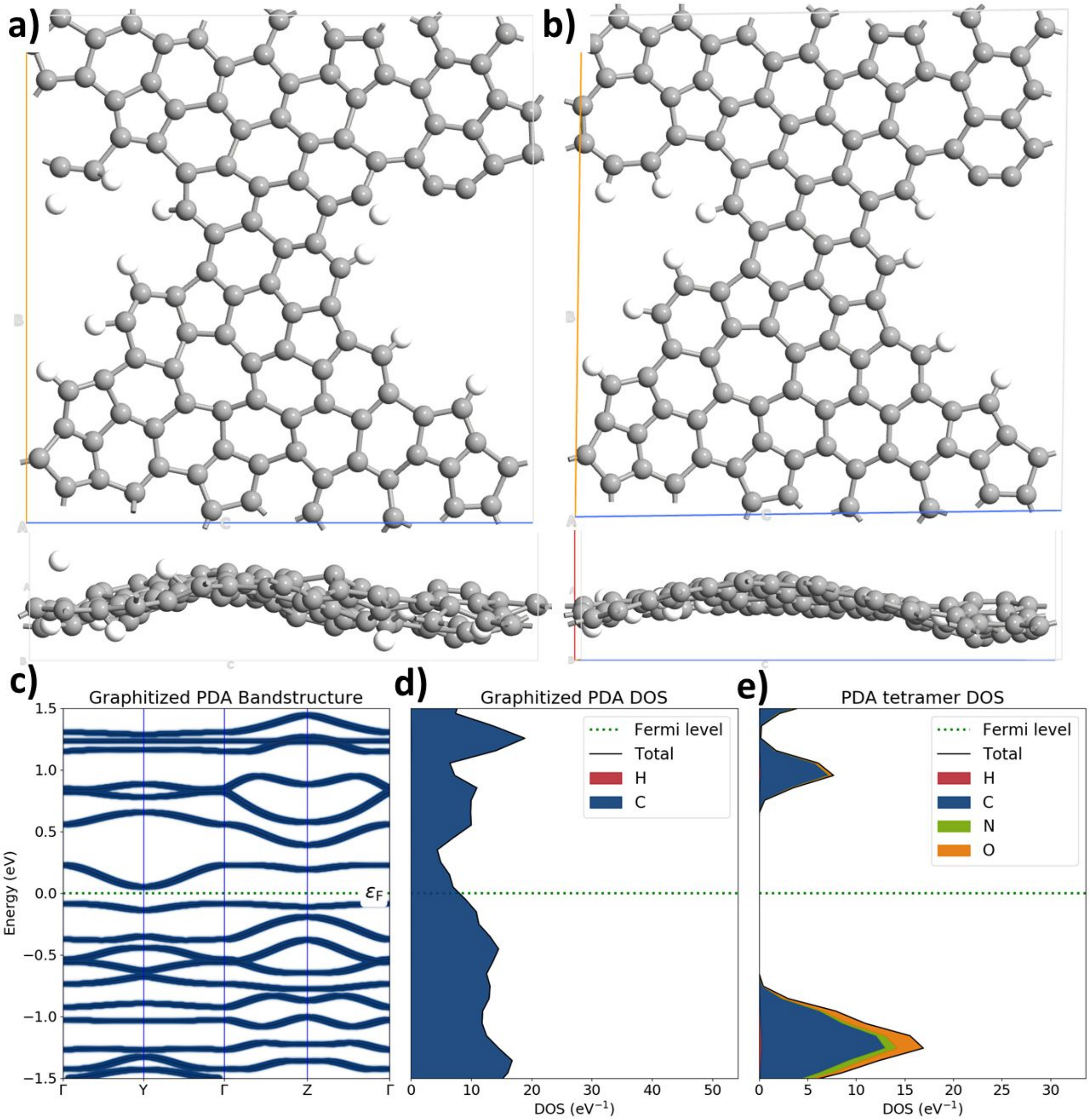

Figure S14. Electronic structure of the graphitized PDA; a) lgPDA sheet taken from ReaxFF MD simulation, b) DFT-optimized geometry of the resulting sheet; c-d) surface bandstructure and density of states of the sheet; e) density of states of single DHI tetramer.

Supplementary serotonin sensing measurements

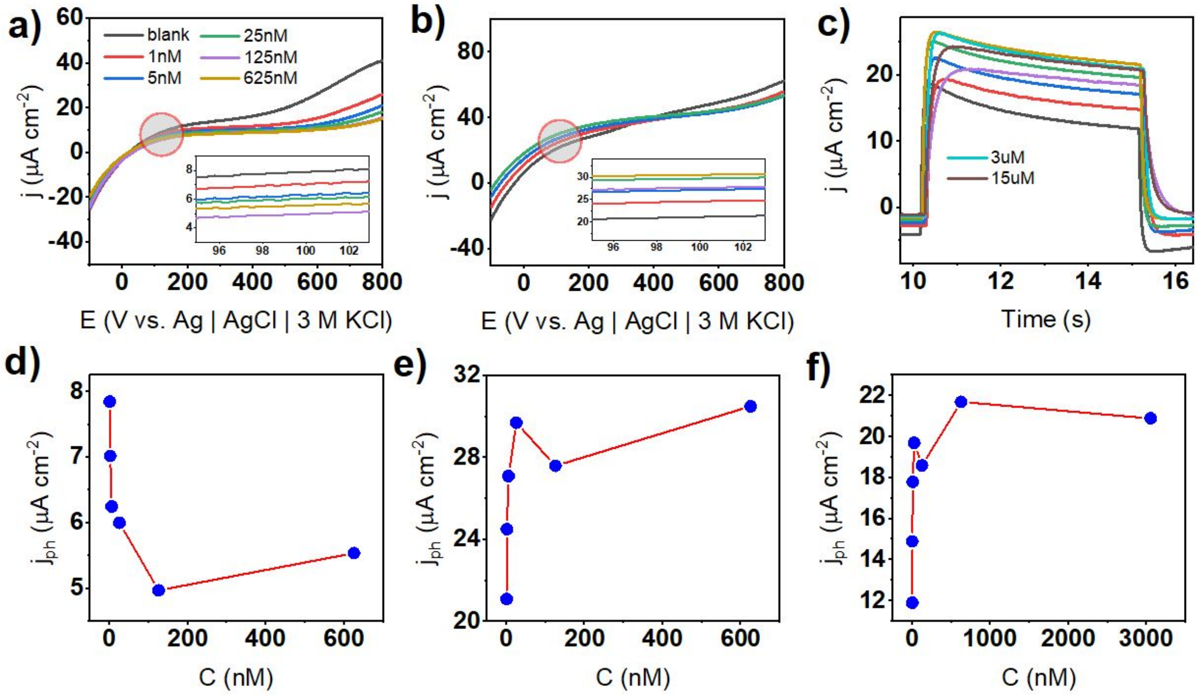

Figure S15. Supplementary PEC serotonin sensing measurements using TNT_lgPDA_365; a) LSV curves in dark conditions, b) LSV curves during illumination, c) chopped CA registered for higher concentrations (up to 15 μM); d-f) are respective calibration curves. The light source and electrolyte are the same as in other PEC experiments (Figure 10).

References

(1) Vargas, W. E.; Niklasson, G. A. Applicability Conditions of the Kubelka–Munk Theory. *Appl. Opt.* **1997**, *36* (22), 5580. https://doi.org/10.1364/AO.36.005580.

(2) Smidstrup, S.; Markussen, T.; Vancraeyveld, P.; Wellendorff, J.; Schneider, J.; Gunst, T.; Verstichel, B.; Stradi, D.; Khomyakov, P. A.; Vej-Hansen, U. G.; Lee, M.-E.; Chill, S. T.; Rasmussen, F.; Penazzi, G.; Corsetti, F.; Ojanperä, A.; Jensen, K.; Palsgaard, M. L. N.; Martinez, U.; Blom, A.; Brandbyge, M.; Stokbro, K. QuantumATK: An Integrated Platform of Electronic and Atomic-Scale Modelling Tools. *J. Phys. Condens. Matter* **2020**, *32* (1), 015901. https://doi.org/10.1088/1361-648X/ab4007.

(3) Smidstrup, S.; Stradi, D.; Wellendorff, J.; Wellendorff, J.; Khomyakov, P.; Vej-Hansen, U. G.; Ulrik G. Vej-Hansen; Maeng Eun Lee; Maeng-Eun Lee; Maeng Eun Lee; Lee, M.-E.; Ghosh, T. K.; Jónsson, E. Ö.; Jónsson, H.; Stokbro, K. First-Principles Green's-Function Method for Surface Calculations: A Pseudopotential Localized Basis Set Approach. *Phys. Rev. B* **2017**, *96* (19), 195309. https://doi.org/10.1103/physrevb.96.195309.
